# Antimicrobial Activity of Spices Popularly Used in Mexico against Urinary Tract Infections

**DOI:** 10.3390/antibiotics12020325

**Published:** 2023-02-03

**Authors:** Ariadna Jazmín Ortega-Lozano, Estefani Yaquelin Hernández-Cruz, Tania Gómez-Sierra, José Pedraza-Chaverri

**Affiliations:** 1 Department of Biology, Faculty of Chemistry, National Autonomous University of Mexico (UNAM), Mexico City 04510, Mexico; 2 Postgraduate in Biological Sciences, National Autonomous University of Mexico, University City, Mexico City 04510, Mexico

**Keywords:** urinary tract infections (UTIs), uropathogens, spices, antimicrobial activity

## Abstract

Urinary tract infections (UTIs) are the most common infectious diseases worldwide. These infections are common in all people; however, they are more prevalent in women than in men. The main microorganism that causes 80–90% of UTIs is *Escherichia coli*. However, other bacteria such as *Staphylococcus aureus, Enterococcus faecalis, Pseudomonas aeruginosa, Proteus mirabilis*, and *Klebsiella pneumoniae* cause UTIs, and antibiotics are required to treat them. However, UTI treatment can be complicated by antibiotic resistance and biofilm formation. Therefore, medicinal plants, such as spices generally added to foods, can be a therapeutic alternative due to the variety of phytochemicals such as polyphenols, saponins, alkaloids, and terpenes present in their extracts that exert antimicrobial activity. Essential oils extracted from spices have been used to demonstrate their antimicrobial efficacy against strains of pathogens isolated from UTI patients and their synergistic effect with antibiotics. This article summarizes relevant findings on the antimicrobial activity of cinnamon, clove, cumin, oregano, pepper, and rosemary, spices popularly used in Mexico against the uropathogens responsible for UTIs.

## 1. Introduction

Urinary tract infections (UTIs) are among the most common bacterial infections. Worldwide, in 2019, it was estimated that 404.61 million cases and 236,790 deaths were associated with UTIs [1]. Regarding Mexico, the picture is similar, UTIs are one of the most common pathologies, and approximately 4 million cases are reported each year, and it is the third most common cause of morbidity [2]. UTIs can affect both males and females, but the prevalence is higher in females (>70%) due to the very close presence of the urethra to the anus and the gastrointestinal colonization of pathogens in the vagina [3]. UTIs are an inflammatory response of the urothelium to bacterial infection, and it involves pyelonephritis (kidney infection), urethritis (ureters infection), cystitis (bladder infection), and prostatitis [3]. UTIs are caused by bacteria, viruses, and yeast, although bacteria cause more than 85% of infections. Among the bacteria most commonly associated with UTIs are *Escherichia coli*, *Staphylococcus aureus*, *Klebsiella pneumoniae, Enterococcus faecalis, Pseudomonas aeruginosa, Proteus mirabilis, Acinetobacter baumannii*, and *Staphylococcus saprophyticus* [4]. It is worth mentioning that UTIs are considered established when the pathogen can enter the urinary tract system and reach more than 10^5^ colony/mL in the urine [4]. *E. coli* and *Enterococcus* spp. are the most frequent bacteria in UTIs. Both bacteria are present in the gastrointestinal habitat, which favors acquiring resistance genes from other commensal organisms [5]. Bacterial resistance and recurrence are due to the misuse and abuse of antibiotics and the adherence capacity of uropathogens that allows them to internalize in target cells, forming biofilm [2,6,7]. Furthermore, catheters are estimated to be one of the most common causes of healthcare-associated infection, as it is known that bacteria can form biofilms on catheters, making the control of bacterial biofilm formation an urgent need [8,9].

**Figure 1:**
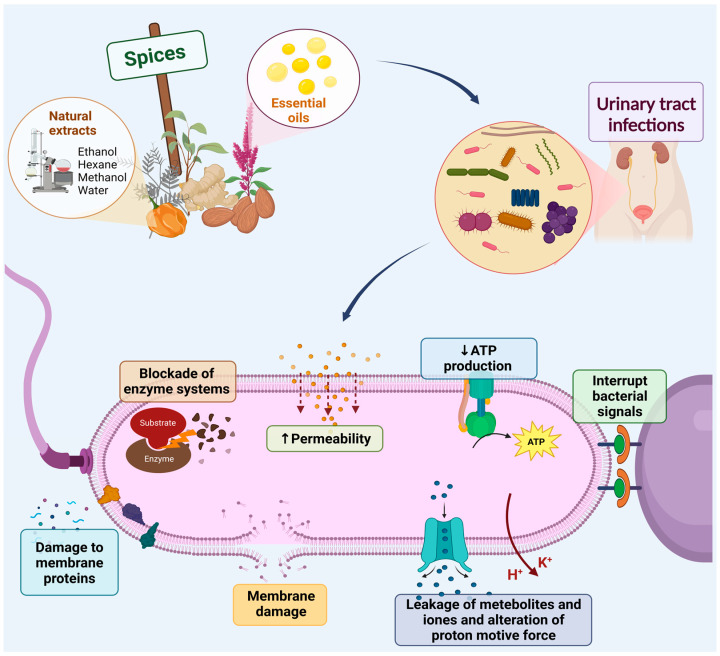
Antibacterial effects of essential oils (EOs). EOs kill bacteria by directly damaging cell membrane lipids and proteins, blocking enzyme systems, altering pH gradient and proton-motive force electrical potential, increasing permeability, disrupting bacterial signaling, and decreasing the adenosine triphosphate (ATP). H^+^: hydrogen; K^+^: potassium. Created with Biorender.com.

As mentioned above, UTIs are usually treated with tetracycline, ampicillin, ciprofloxacin, gentamycin, and amikacin. However, in recent decades we have faced the problem of increasing resistance of bacteria to drugs due to repeated antibiotic therapies, leading to an increase in recurrent infections and the difficulty of treatment and prophylaxis [10,11]. Due to this problem, interest in natural remedies has increased; medicinal plants and mushrooms represent an essential part of traditional medicine and have been used throughout history as treatments for different pathologies. This effect of medicinal plants is because they contain phytochemicals that can treat disease. These phytochemicals can be obtained by preparing plant extracts or essential oils (EOs) [12,13]. Plant extracts are multicomponent mixtures of active, partially active, and inactive substances. Plant extracts can be obtained using one or several solvents, noting that the solution obtained can be cooled and flow and that the solvent or solvents are totally or partially eliminated. The choice of solvent(s) for the extraction of biomolecules from plants is based on the polarity of the solute of interest [14]. Therefore, the composition of plant extracts depends on the method of preparation and the plant materials used [13,15]. The extraction of EOs can be performed by steam or dry distillation, mechanical treatment, fermentation, crushing, hydrolysis, and airing [16,17]. EOs are volatile, odorous substances with different degrees of antimicrobial activity. The EOs with the highest antibacterial activity are characterized by a high content of phenolic compounds, such as carvacrol, eugenol, and thymol, which cause alterations in the cell membrane [16]. These properties of plant-derived compounds depend on the plant species, climatic conditions, soil composition, cultivation methods or harvesting areas, extraction method, and solvent composition [16,17]. Studies have reported that antimicrobial phytochemicals can directly damage the cell membrane of bacteria, causing cell membrane rupture, blockage of enzyme systems, disruption of ion exchange, alterations of pH gradient, and proton motive force electric potential (Figure 1) [12,16].

## 2. Spices

Spices are aromatic plants widely used in Mexico to flavor, color, or preserve food. In particular, the Food and Drug Administration (FDA) Organization defined *spices* as: “whole, broken or ground aromatic vegetable substances, whose significant function in food is to flavor rather than to nourish” [18]. Spices usually come from the dried part of a plant, such as flower buds, flowers (cloves, saffron); bark (cinnamon); root (ginger, turmeric); fruits/berries (cloves, chili, black pepper); or seeds (cumin) [19]. Additionally, some herbs are included in the spices, such as marjoram, parsley, mint, rosemary, oregano, and thyme [19].

The ISO 676 document lists about 109 species of aromatic plants and spices used in food, which can be classified in different ways [20]; the taxonomic classification divides them into monocotyledons (embryo with a single cotyledon), which includes garlic, ginger, turmeric, and herbs, or dicotyledons (embryo with two cotyledons), such as paprika, pepper, nutmeg, cinnamon, and cloves [21]. They can also be classified according to their organoleptic properties in hot spices such as chili, pepper, and ginger; mild spices such as paprika and coriander; aromatic spices such as cinnamon, cloves, cardamom, and cumin; herbs such as basil, bay leaves, dill leaves, marjoram, tarragon, and thyme; and in aromatic vegetables such as onion, garlic, shallot, and celery [20]. Alternatively, according to their use, they are classified as fresh (herbs), dried, or processed (extracts, oleoresins, and resins) [22].

Due to its properties and wide range of applications, the spice trade has become an important economic activity. In 2020, spices were widely traded worldwide for a total of USD 3.61 billion. Between 2019 and 2020, spice exports grew by 23.2%. The spice trade represents 0.022% of total world trade [23]. Furthermore, due to the globalization of food production and trade, almost all products are available year-round in developed countries [24].

Research on its health benefits has increased significantly in recent decades, as many spices are known to have properties that reduce the risk of chronic disease. It has been reported that spices can protect against cardiovascular diseases, neurodegenerative diseases, chronic inflammation, cancer, obesity, and type 2 diabetes [25,26,27,28]. In addition, the use of spices in food reduces the use of salt as a flavoring agent; that is, it causes reduced sodium intake, which has additional benefits for cardiovascular health [29]. Spices also have antibacterial, antiviral, and antifungal properties [30]. Therefore, they are widely used as food preservatives [31]. Additionally, it has been reported that spices represent an alternative to inhibit the bacteria responsible for UTIs [32,33].

Most of the beneficial health properties of spices are mediated through the direct action of their phytochemicals, especially polyphenols or polyphenol degradation products. These phytochemicals have broad antioxidant properties and target specific receptors or enzymes involved in various anti-inflammatory pathways or immune responses [19]. Phenolic acids and flavonoids, especially flavones and flavonoids, are spices’ predominant class of polyphenols [34]. Furthermore, the antimicrobial properties of spices are attributed to their unique volatile oils and oleoresins [35]. Because spices are obtained from aromatic plants and herbs, they are generally considered safe (GRAS) [36].

Mexico is one of the main spice-producing areas [20]. Its gastronomy is defined by being varied, spicy, tasty, or seasoned. Mexican food comprises a wide variety of dishes and culinary techniques, with flavors originating in pre-Hispanic times and influenced by the cuisine of other cultures. Spices, however, are one of the repetitive ingredients in each national dish. Because of them and their combination of aromas, colors, textures, and flavors, the history of Mexican cuisine has gained international recognition. UNESCO declared Mexican gastronomy the Intangible Heritage of Humanity in November 2010 [37,38].

This review highlights the antimicrobial activity of spices popularly used in Mexico, such as cinnamon, cloves, cumin, oregano, pepper, and rosemary, in UTIs.

## 3. Cinnamon

The trees and shrubs of the Cinnamomum genus belong to the Lauraceae family and contain around 300 species worldwide. The most common are *C. burmanni, C. camphora, C. cassia, C. osmophloeum, C. verum,* and *C. zeylanicum* [39,40,41,42]. The fruit, leaf, and bark of the cinnamon tree contain bioactive compounds such as cinnamyl acetate, coumarin, eugenol, eucalyptol, *trans*-cinnamaldehyde, L-borneol, caryophyllene oxide, benzoic acid, linalool, caffeic acid, and camphor [40,42,43,44]. Cinnamon leaves and the bark have been reported to have antioxidant activity [41]. However, the peeled and dried bark is a popular spice used as a condiment and flavoring agent in desserts, spicy sweets, tea, liqueurs, cereals, bread, fruit, and chocolates. Mexico is the biggest importer of *C. zeylanicum*, also known as true cinnamon [45], and it is also used in chocolate production and sweet and savory dishes. Moreover, cinnamon is used in traditional medicine worldwide due to its antimicrobial, antifungal, nematicidal, antipyretic, insecticidal, antioxidant, and antidiabetic properties [40,41,43,46,47,48]. Although cinnamon is recognized as a safe spice, with a tolerable daily intake of 0.1 mg/kg/day, adverse effects such as stomach and bowel disorders and allergic reactions have been described [45,49].

The solvents used to extract the bioactive compounds of cinnamon are water, methanol, ethanol, and chloroform [44]. Hydrodistillation, steam distillation, Soxhlet extraction, and maceration are the most common methods to prepare cinnamon extract oils from leaf, fruit, and bark. Nevertheless, novel extraction methods such as supercritical fluid, assisted by microwave radiation, superheated water, supercritical carbon dioxide (CO_2_), or ultrasounds have been developed [44,46,50]. The EOs are obtained from bark through hydrodistillation, steam distillation, and supercritical fluid extraction techniques. EOs extracted contain trans-cinnamaldehyde, eugenol, cinnamyl acetate, camphor, and linalool, representing about 85% of the total oil composition [46,51]. However, *trans*-cinnamaldehyde is an unstable compound that can be oxidized to cinnamic acid when exposed to air, losing the acrolein group, which is responsible for antimicrobial activity. Nevertheless, the other bioactive compounds in cinnamon EO have antimicrobial activity and synergistic or additive effects with *trans*-cinnamaldehyde [40,52,53].

The bioactive compounds in cinnamon EO, such as *trans*-cinnamaldehyde, pass through bacteria cell walls, altering the permeability and integrity of the membrane and, consequently, causing loss of transporting proteins, metabolites, and ions which leads to cytoplasmic coagulation and denaturation of proteins [40,54,55,56]. Moreover, *trans*-cinnamaldehyde downregulated the F1F0-ATPase complex, inducing the depletion of intracellular adenosine triphosphate (ATP) synthesis and growth rate. Furthermore, *trans*-cinnamaldehyde causes energy deprivation, amino acid decarboxylation activity inhibition within the cells, and cell death [40,55]. The antimicrobial activity of cinnamon bark extracts is shown in Table 1.

It has been described that the EO of *C. cassia* obtained by hydrodistillation has a minimum inhibitory concentration (MIC) against bacterial strains collected from patients with UTIs, such as *E. coli* in the range of 0.30 to 2.50 mg/mL, *P. aeruginosa* to 2.5 to 5.0 mg/mL, *P. mirabilis* 0.30 to 1.25 mg/mL, and *K. pneumoniae* to 0.16 to 31 mg/mL. In addition, this EO has inhibition zones around 12 to 39 mm for Gram-negative bacteria strains [57].

Moreover, cinnamon bark oil (*C. zeylanicum*) has inhibitory and bactericidal activity against *P. aeruginosa* and its multidrug-resistant strains isolated from patients with UTIs. The MIC and minimal bactericidal concentrations (MBC) values are in the range of 0.1125 to 0.225% (v/v) and 0.1125 to 1.8%, respectively. Moreover, cinnamon bark oil has a synergistic interaction with some antibiotics. The main bioactive compounds identified in this cinnamon bark oil are *trans*-cinnamaldehyde and eugenol. However, *trans*-cinnamaldehyde possessed better antimicrobial activity to reduce 6 log CFU/mL to this pathogen [56].

In addition, ethanolic extract of *C. zeylanicum* could inhibit the growth of colonies of *E. coli*, *P. aeruginosa*, and *K. pneumoniae* obtained from urine samples of patients with UTIs. The results indicate that the mean zone of inhibition against the pathogens mentioned before is 11.72 mm, 20.16 mm, and 25.50 mm, respectively. This inhibition zone is similar to norfloxacin, a common antibiotic for treating UTIs. Although *trans*-cinnamaldehyde has been identified as the bioactive antimicrobial compound in the cinnamon extract, it was found that the zone of inhibition of *trans*-cinnamaldehyde against *P. aeruginosa* (14.82 mm) and *K. pneumoniae* (18.45 mm) was lower than the of the whole extract, while against *E. coli* (24.39 mm), the zone of inhibition was higher compared to cinnamon extract against [58]. As mentioned above, this compound could have antimicrobial activity and be enhanced by other bioactive compounds in the cinnamon extract. Because of this, the isolated bioactive compound *trans*-cinnamaldehyde was used in a uropathogenic *E. coli* colonization in C57BL/6 female mice to reproduce a UTI. Female mice were supplemented in the feed with *trans*-cinnamaldehyde at 0.1, 0.2, and 0.4% for 14 days. On day 10, mice were infected with *E. coli* through transurethral inoculation. It is observed that *trans*-cinnamaldehyde reduced pathogen populations in the bladder and urethra in a concentration-dependent manner [59].

Moreover, it is described that aqueous *C. zeylanicum* extract (0.1 g/mL) has antimicrobial activity against isolated pathogens isolated from the urine of patients with UTIs such as *E. coli*, *K. pneumoniae*, *S. aureus*, *Enterobacter spp*, *P. aeruginosa*, *S. typhi*, and *S. flexneri*.

Furthermore, this aqueous cinnamon extract has moderate antifungal activity against *Aspergillus niger* and *Candida albicans* compared to ketoconazole. Nevertheless, due to the significant extraction of *trans*-cinnamaldehyde, the EO cinnamon extract has more antifungal activity than the aqueous extract [55]. Finally, the EO of cinnamon has MIC and MCB values of 0.125% and 0.25%, respectively, against *K. pneumoniae* isolated from patient urine samples and has good efficacy against this biofilm produced by this Gram-negative bacterium [60].

**Table 1:** Antimicrobial activity of cinnamon bark extracts.

Cinnamon Specie	Type of Extract	Phytochemicals	Uropathogen	MIC	MBC	Diameter of the Inhibition Zone (mm)	Ref.
*C. cassia*	Essential oil	*trans*-cinnamaldehyde, cinnamic acid, eugenol, benzaldehyde	*E. coli*	26–35 mg/mL	ND	26–38	[57]
			*P. aeuruginosa*	12–19 mg/mL	ND	12–19	
			*P. mirabilis*	30–39 mg/mL	ND	30–39	
			*K. pneumoniae*	27–32 mg/mL	ND	27–32	
*C. zeylanicum*	Essential oil	*trans*-cinnamaldehyde, cinnamic acid, eugenol, benzaldehyde	*P. aeuruginosa*	0.11–0.2%	0.1125–1.8%	ND	[56]
NS	Essential oil	*trans*-cinnamaldehyde, cinnamic acid, eugenol, benzaldehyde	*E. coli*	1 mg/mL	4 mg/mL	19.2	[54]
			*S. aureus*	1 mg/mL	2 mg/mL	28.7	
*C. verum*	Essential oil	*trans*-cinnamaldehyde, cinnamic acid, eugenol	*K. pneumoniae*	0.125%	0.25%	ND	[60]
*C. zeylanicum*	Ethanolic extract	Tannins, Flavonoids, anthraquinones, saponins	*E. coli*	ND	ND	11.72	[58]
			*K. pneumoniae*	ND	ND	25.50	
			*P. aeuruginosa*	ND	ND	23.25	
*C. verum*	Ethanolic extract	Tannins, Flavonoids, anthraquinones, saponins	*P. aeuruginosa*	10 mg/mL	20 mg/mL	12.3	[61]
			*K. pneumoniae*	20 mg/mL	40 mg/mL	15.3	
			*S. aureus*	10 mg/mL	20 mg/mL	12.5	
	Dichloromethane extract	Flavonoids, anthraquinones, alkaloids, saponins	*P. aeuruginosa*	20 mg/mL	40 mg/mL	10.0	
			*K. pneumoniae*	20 mg/mL	40 mg/mL	12.3	
			*S. aureus*	5 mg/mL	10 mg/mL	11.5	
	Hexane extract	Tannins, alkaloids, flavonoids, anthraquinones, saponins	*P. aeuruginosa*	10 mg/mL	20 mg/mL	10.5	
			*K. pneumoniae*	20 mg/mL	20 mg/mL	14.5	
			*S. aureus*	5 mg/mL	10 mg/mL	15.0	

ND: not determined; NS: not specified in the article. MIC: minimum inhibitory concentration; MBC: minimum bactericidal concentration.

## 4. Clove

Clove (*Syzygium aromaticum, syn. Eugenia caryophyllata*) is a plant used for centuries as a food preservative and for many medicinal purposes. It belongs to the family of Myrtaceae, the subfamily Myrtoideae, and the tribe Syzygieae [62]. It is native to Maluku Island in Indonesia. However, today it is cultivated in various parts of the world, especially in countries with tropical and subtropical environments, such as Indonesia, Sri Lanka, India, Tanzania, Malaysia, Madagascar, and Pakistan [47]. Its dried buttons (flowers that have not yet opened) are called cloves or gyrofles and are used as a spice in kitchens worldwide [63]. In Mexican cuisine, cloves are widely used to season sweet and salty foods. In salty stews, it is included whole or ground in moles, marinades, and other dishes. As for desserts, it is used above all in fruit syrups such as guava candy.

Cloves are a valuable source of phenolic compounds such as glycosides, flavonoids (kaempferol and quercetin), saponins, tannins, and EOs. Gallic acid is the phenolic acid with the highest concentration (783.50 mg/100 g fresh weight), although we also find caffeic, ferulic, and salicylic acids in smaller quantities [64]. However, the main bioactive component of cloves is eugenol [2-methoxy-4-(2-propenyl) phenol], an allyl chain substituted guaiac that is a member of the allylbenzene class of chemical compounds [65], and is present in concentrations ranging from 9381.70 to 14,650.00 mg/100 g fresh plant weight [64].

In medicine, various therapeutic properties have been attributed to clove, including antifungal, antibacterial, antiviral, anti-inflammatory, antioxidant, hepatoprotective, antistress, antidiabetic, antinociceptive, anesthetic, and even anticancer activities [63]. Clove has also been widely used to treat UTIs due to its wide range of antimicrobial activities against Gram-positive and Gram-negative bacteria [66,67,68,69,70]. Table 2 shows the antimicrobial activity of the different clove extracts.

Among the most used clove extracts to treat UTIs is clove oil, an aromatic oil extracted from the buds and leaves of *S. aromaticum* trees. It is traditionally obtained by hydrodistillation, steam distillation, or solvent extraction. These processes are inexpensive but can induce thermal degradation, hydrolysis, and water solubilization of some fragrance components [71,72]. Some authors have also carried out clove oil extraction with CO_2_, which offers significant advantages over traditional methods. For example, the higher percentage of eugenol active antioxidant ingredients and a shorter extraction time, among others [71]. 

Clove oil has been highly successful against the bacteria responsible for UTIs. For example, it has been reported that after 8 h of treatment with clove oil, the population of *E. coli* and *S. aureus* is reduced by 99.999% and 99.9999%, respectively [67]. Additionally, in a study conducted with 60 clinical isolates from the urine of UTIs patients, clove EO was found to be more effective against *E. coli* strains than against *K. pneumoniae* strains. This is because the growth inhibition of *E. coli* was 24.5 (22.75–28) mm, while for *K. pneumoniae* it was only 22 (20–24) mm [66]. In fact, clove oil has been reported to kill *E. coli* resistant to recommended antibiotics [73].

The efficacy of EOs differs from one type of oil to another and from the target bacteria, depending on their structure (Gram-positive and Gram-negative bacteria) [74,75]. However, in a study comparing clove oil with cinnamon, bell pepper, thyme, oregano, and rosemary oils, clove oil was reported to be the most effective in inhibiting the growth of *S. typhi*, *S. aureus*, and *P. aeruginosa* [76]. Furthermore, it has been observed that mixing clove oil vapor with cinnamon oil vapor antagonistically inhibits the growth of *E. coli*. In contrast, both oils exerted a synergistic effect for inhibiting *Listeria monocytogeneses*, *Bacillus cereus*, and *Yersinia enterocolitica*, when the maximum inhibition concentrations are used [77].

So far, there are few studies on the antibacterial mechanism of clove oil, especially at the molecular level. However, previous studies report that clove oil could be destroying the integrity of cell membranes, which triggers the egress of biological macromolecules and intracellular enzymes and interferes with protein synthesis [78,79]. Clove oil may also affect bacteria by slowing respiratory metabolism. Clove oil treatment has decreased the intracellular ATP of *E. coli* and *S. aureus* by up to 76.23% and 71.55%, respectively. In addition, in this same study, a decrease in nucleic acids was observed, indicating that clove oil affects the permeability of the cell membrane [67]. In another study on *L. monocytogeneses*, clove oil was reported to reduce the activity of three key enzymes (isocitrate dehydrogenase, citrate synthase, and α-ketoglutarate dehydrogenase) in the citric acid cycle pathway, affecting the content of metabolites in the pathway. In addition, it has been shown that eugenol, the main component of clove oil, can change the structure of DNA through the formation of eugenol-DNA chimeras [67].

Another preparation that has been used against UTIs is the phytochemical extraction of the clove spice, which can be carried out by aqueous-ethanolic maceration. Previous studies have shown that 80% ethanol effectively extracts most bioactive phytochemical compounds, especially flavonoids [80]. In fact, a study comparing extraction with different solvents in spices reported that the 80% ethanol extraction method has the highest inhibition towards Gram-negative and Gram-positive bacteria [81].

To prepare the ethanolic extract of cloves, the spices are washed with sterile water and subsequently dried at 40 °C. Then, the clove is ground with a blender to a fine powder. Finally, a maceration with 80% ethanol is carried out, and the filtrate is concentrated using a rotary evaporator [82,83].

A recent study demonstrated that ethanolic extract of clove (2000 μg) exhibited broad-spectrum inhibition against Gram-negative and Gram-positive UTIs-causing pathogens: *P. mirabilis* (19.7 mm), *Staphylococcus epidermidis* (18 mm), *S. aureus* (14.7 mm), *E. coli* (12.7 mm), *K. pneumoniae* (12.3 mm) (depending on the size of the inhibition halo) [83]. Interestingly, the comparison between ethanolic clove extract and commercial clove EO revealed that the former demonstrated more potent antimicrobial and antioxidant properties at a similar concentration of eugenol. However, Gas Chromatography/Mass Spectrometry (GC/MS) indicates that the ethanolic extract has a lower concentration of eugenol than the EO, suggesting that eugenol is not the only compound responsible for the antimicrobial effect observed for the ethanolic extract [83].

In another study where 221 Gram-negative bacteria were isolated and the production of b-lactamases, enzymes responsible for resistance to b-lactam antibiotics, was analyzed, it was reported that the ethanolic extract of cloves was effective against all Gram-negative isolates. However, the best antibacterial activity was shown against *P. mirabilis* species with an inhibition halo of 19 mm. Likewise, it was found that the ethanolic extract of cloves has a different antibacterial potential depending on the Gram-negative uropathogenic [68].

In another study, aqueous and ethanolic extracts of clove, cinnamon, and garlic were prepared and tested for their antibacterial efficacy against isolated *E. coli*. Clove extracts were shown to have one of the best antibacterial activities against UTIs strains according to their mean values of the zone of inhibition (13.33 mm). The combined effect of 10% plant extracts with antibiotics such as resistance drugs ampicillin, imipenem, ciprofloxacin, norfloxacin, and nalidixic acid was also tested and showed a pattern of susceptibility with increasing inhibition zone diameter in three UTIs strains [5]. Therefore, we could say that the use of ethanolic clove extract to treat UTIs could be complementary to the use of antibiotics due to its additive effect with them.

There are no studies on the antibacterial mechanism of the ethanolic extract of clove, although it could be suspected that it acts the same or similar to the EO. Until now, the antibacterial activity of the ethanolic extract has only been attributed to its antioxidant properties. A recent study reported that the EC_50_ of DPPH (1,1-diphenyl-2-picrylhydrazyl), ABTS (2,2′-azino-bis(3-ethylbenzothiazoline-6-sulfonic acid)), and reducing power assay for the ethanolic extract content of clove was 0.037 mg/mL, 0.68 mg/mL, and 0.44 mg/mL, respectively [83].

Finally, it is essential to mention that the consumption of cloves has been declared safe for humans. Clove crude EO is classified as GRAS by the US Food and Drug Administration [84]. Acute and chronic toxicity studies with clove oil conclude that there are no adverse effects in albino rats [85]. Additionally, an aqueous extract of dried clove buds (known as ‘Clovinol’), rich in polyphenols, was found to be safe in rats [86]. Furthermore, eugenol is rapidly absorbed, metabolized in the liver, and eliminated within 24 h when consumed orally [87]. However, eugenol can be toxic in children under two years of age in relatively small amounts (5–10 mL) [88]. Additionally, if cloves are consumed or used excessively topically or by an allergic person may experience side effects. For example, oral intake of cloves may cause lactic acidosis, nausea, numbness, dizziness, or tiredness [89]. Furthermore, it can lead to liver problems associated with stomach pain, clay-colored stools, dark urine, and sometimes jaundice [90]. If applied topically, it could cause itchy rashes with mild skin irritation, swollen or bleeding gums, erection problems, and delayed ejaculation [89]. Previously, the World Health Organization (WHO) set the daily human intake of clove oil at 2.5 mg/kg body weight for humans [91].

In short, clove extracts can be used to develop a new antimicrobial drug that is the need of the hour. However, more research is required on the mechanisms of action, identification, and characterization of bioactive molecules, particularly their antibacterial activities in vivo against human pathogens.

**Table 2:** Antimicrobial activity of *S. aromaticum* extracts.

Type of Extract	Phytochemicals	Uropathogen	MIC	MBC	Diameter of the Inhibition Zone (mm)	Ref.
Clove oil	Eugenol, b-caryophyllene, vanillin, crategolic acid, bicornin, galotanic acid, methyl salicylate eugenin, kaempferol, ramnetin and eugenitin, oleanolic acid, stigmasterol, campesterol, and various sesquiterpenes	*E. coli*	0.5 mg/mL	0.5 mg/mL	ND	[67]
		*S. aureus*	0.5 mg/mL	0.5 mg/mL	ND	
Clove oil	Eugenol, b-caryophyllene, vanillin, crategolic acid, bicornin, galotanic acid, methyl salicylate eugenin, kaempferol, ramnetin and eugenitin, oleanolic acid, stigmasterol, campesterol, and various sesquiterpenes	*A. baumanni*	ND	ND	28	[92]
		*P. aeruginosa*	ND	ND	17	
		*E. faecalis*	ND	ND	25	
		*S. aureus*	ND	ND	20	
Clove oil	Eugenol, b-caryophyllene, vanillin, crategolic acid, bicornin, galotanic acid, methyl salicylate eugenin, kaempferol, ramnetin and eugenitin, oleanolic acid, stigmasterol, campesterol, and various sesquiterpenes	*E. coli* isolated from UTIs patients	2.1 to 3.1 mg/mL	3.1 to 4.2 mg/mL	ND	[73]
		Antibiotic-resistant *E. coli*	2.6 mg/mL	3.7 mg/mL	ND	
Clove oil	Eugenol, b-caryophyllene, vanillin, crategolic acid, bicornin, galotanic acid, methyl salicylate eugenin, kaempferol, ramnetin and eugenitin, oleanolic acid, stigmasterol, campesterol, and various sesquiterpenes	*E. coli* isolated from UTIs patients	5.5 μL/mL and 0.55 μL/mL *	ND	24.5 mm	[66]
		*K. pneumoniae* isolated from UTIs patients	5.5 μL/mL and 0.55 μL/mL *	ND	22 mm	
Ethanolic extract	Eugenol, glycosides, flavonoids, saponins, tannins, and essential oils.	*S. aureus*	5 mg/mL	10 mg/mL	11.4	[93]
		*P. aeruginosa*	5 mg/mL	12.5 mg/mL	9.2	
Ethanolic extract	Eugenol, glycosides, flavonoids, saponins, tannins, and essential oils.	*E. coli*	0.39 mg/mL	0.19 mg/mL	17	[68]
		*K. pneumoniae*	0.78 mg/mL	0.39 mg/ml	16	
		*Enterobacter* species	0.78 mg/mL	0.39 mg/mL	17	
		*Citrobacter* Species	0.39 mg/mL	0.19 mg/mL	18	
		*P. mirabilis*	0.39 mg/mL	0.19 mg/mL	19	
		*P. aeruginosa*	1.56 mg/mL	0.78 mg/mL	14	
		*A. baumanni*	0.78 mg/mL	0.39 mg/mL	18	
Ethanolic extract	Eugenol, glycosides, flavonoids, saponins, tannins, and essential oils.	*P. mirabilis*	ND	ND	19.7	[83]
		*S. epidermidis*	ND	ND	18	
		*K. pneumoniae*	ND	ND	12.3	
		*E. coli*	ND	ND	12.7	
		*S. aureus*	ND	ND	14.7	

* Depending on the strain. ND: not determined; MIC: minimum inhibitory concentration; MBC: minimum bactericidal concentration.

## 5. Cumin

Cumin (*Cuminum cyminum L*.) is an aromatic plant that belongs to the Apiaceae family, Apioideae subfamily, Scandiceae tribe, and Daucinae subtribe [94]. It has a branched and ribbed stem, leaves divided into filaments and strongly lobed, small white or pink flowers, and ovate or spindle-shaped fruits, light brown or light grey. The seeds are ovoid, and two grains are connected in one body; one side is raised and ribbed, and the other is flat, brown in color, fragrant in odor, and pungent in taste [95]. The seeds are mainly used as a seasoning or flavoring agent for different culinary purposes [96]. The seed’s EO is used in abundance to make soups, stews, sausages, cheeses, pickles, curries, meats, and chutneys. Furthermore, the seeds are widely used in the perfume industry due to their intense aroma [97]. Additionally, it is used in medical preparations such as toothpaste, mouthwashes, and soaps [96].

Today, cumin is produced in countries such as Chile, Mexico, Syria, Egypt, Morocco, Turkey, Iran, Tajikistan, Uzbekistan, and China, where India accounts for 70% of world production and 90% of consumption [98]. Particularly in Mexico, it was popularized by the Spanish, who traditionally used it to make blood sausage and other dishes, and it quickly became part of the local cuisine due to its spicy flavor [99].

Cumin seed contains 22.27 to 23.80% total lipids and 2.4 to 5.0% EO (volatile). It also has organic acids such as aspartic, benzoic, citric, malic, propionic, tartaric, ascorbic, maleic, oxalic, and fumaric acids, phenols such as salicylic, cinnamic, gallic, p-hydroxybenzoic acid, hydroquinone, resorcinol and flavonoids such as rutin, quercetin, and coumarin [98,100,101]. Limonene, α- and β-pinene, 1, 8-cineole, *o*-and *p*-cymene, α- and γ-terpinene, safranal, and linalool are compounds that are also found in cumin seed. However, cuminaldehyde, cymene, and terpenoids are the main bioactive components, although the EO, in addition to cuminaldehyde, also has paracymene [95,98].

Cumin has different preparations. The most used is the EO extracted from the seeds with the hydrodistillation method. For extraction with this method, the dried and macerated seeds are placed in a distillation apparatus with distilled water for three to four hours. The oil is then extracted and stored in dark vials until use [102,103,104]. The main components of cumin can also be extracted using different solvents such as methanol, acetone, butanol, alcohol, and even water [104,105,106].

In medicine, cumin extracts have been used as an antiseptic, antispasmodic, anticancer, and treatment for digestive disorders, colic, and dyspeptic headaches [107]. In addition, *Cuminum cyminum* seeds have been shown to possess significant biological properties, such as antibacterial and antifungal activity [104]. Cumin has played an important role in UTIs due to its antimicrobial activity against Gram-positive and Gram-negative bacteria [103,104,106]. Table 3 shows the antimicrobial activity of the different cumin extracts.

Cumin EO has potent antimicrobial activity even against strains of *E. coli* resistant to multiple drugs, including tetracycline, erythromycin, amoxicillin, ceftazidime, and cefixime. A study carried out on 12 strains of *E. coli* isolated from the urine of patients hospitalized with UTIs showed that cumin EO had a differential inhibitory effect between the different isolates. Approximately 24.9% of *E. coli* isolates showed low MICs (<50 ppm), while 41.6% had moderate MICs (100 ppm), and 16.6% of the isolates had high MICs (250 ppm) [102]. Likewise, another study observed that cumin oil and methanolic extract have better antibacterial activity in uropathogenic isolates than amoxicillin; however, this did not happen with other antibiotics. In this study, the EO and the methanolic extract were tested against *E. coli*, *K. pneumoniae*, *P. aeruginosa*, *S. agalactiae*, group A *streptococci*, *E. faecalis*, *S. epidermidis*, *S. aureus*, and *S. saprophyticus*, isolated from samples of 95 UTIs patients, but without malignant diseases, diabetes and immunosuppression [104]. In another study carried out in 2016, the effect of cumin oil was compared with chamomile and clove oil. Cumin oil was found to inhibit most bacteria. Furthermore, it was more effective when used with some antibiotics, suggesting that it could be an adjunctive treatment [81].

The mechanism by which cumin EO exerts its antibacterial effects remains unclear. However, it has been reported to cause cell wall damage or changes in outer membrane proteins; these effects could be attributed to the molecular characteristics of the aldehydes present [103,108,109]. It should be noted that bacterial DNA degradation is not a proven antibacterial mechanism for cumin EO. It was previously reported that cumin AE could not induce DNA degradation of the R plasmid of clinical isolates of *K. pneumoniae* [103]. In addition, the aqueous-ethanolic extract (30/70) of cumin has been reported to have significant antibacterial and antioxidant activities [106].

Finally, we must consider that cumin use has side effects, including contact dermatitis, respiratory reactions, and liver cancer (above dietary levels) [110]. Patients with stomach ulcers, liver disorders, and pregnant or lactating women should use cumin with caution. Patients should also be aware of the use of drugs similar to cumin, including antibiotics, anticancer drugs, antifungals, anti-inflammatory drugs, antioxidants, anticonvulsants, cholesterol, and lipid-lowering drugs, estrogen and gastrointestinal drugs, pesticides, iron, morphine, opioids, osteoporosis agents, analgesics and phytoestrogens [95].

In summary, cumin extracts can be used as antibiotics against UTIs-inducing pathogens, even those resistant to drugs. However, it is important to continue updating the information on cumin in combination with antibiotics to enhance the inhibitory effect of uropathogens and study the possible mechanisms behind this protection.

## 6. Oregano

Oregano belongs to the Lamiaceae family and is an aromatic plant cultivated in several regions of the world, whose commercial value is due to its characteristics as a spice, condiment, and medicinal properties. The Greek oregano (*Origanum vulgare*) is the most representative species and has been the most studied oregano species. The *O. vulgare* species comprise several subspecies, *O. vulgare* subsp. L. *vulgare*, *O. vulgare* subsp. L. *glandulosum*, *O. vulgare* subsp. L. *gracile*, and *O. heracleoticum*. In Mexico, at least 40 endemic species are known; it is an important culinary ingredient and has a wide distribution in the arid and semi-arid zones of the country [111,112].

Concerning the medicinal properties of oregano, it has been reported to have antibacterial, antifungal, antiparasitic, antimicrobial, and antioxidant properties [113]. Approximately 100 volatile and non-volatile ingredients have been identified in *O. vulgare*. According to their hydrophilic and hydrophobic characteristic, there are two main phytochemicals in the EO of *O. vulgare* and phenolic compounds (flavonoids and phenolic acids). Other biologically active compounds include terpenoids, tannins, and sterols [114]. The antimicrobial properties of oregano are mainly attributed to thymol and carvacrol, as well as their precursor monoterpenes *p*-cymene and γ-terpinene at a lower proportion [16]. Thymol can inhibit proinflammatory molecules, neutralize free radicals, and be antibacterial, antifungal, antiproliferative, and analgesic. Carvacrol also possesses antibacterial, antifungal, antiviral, immunomodulatory, antiproliferative, antioxidant, and anti-inflammatory activities [115].

Several mechanisms have been proposed to explain the antibacterial activity of EO from *O. vulgare*. Those mechanisms include inhibiting the production or activity of bacterial enzymes (such as lipase and coagulase), efflux pump inhibition, antibiofilm agents, effect on ATP concentration, and effect on the cytoplasmic membrane. In the latter mechanism, the carvacrol can damage the cellular membrane and reduce the cellular membrane pH gradient, leading to the proton motive force, reduction in the ATP pool, and cell death [112,114,116]. The antimicrobial activity of oregano extracts is shown in Table 4.

In a study by Zapién-Chavarría et al. [116], it was demonstrated that two oregano species endemics to Mexico possess antimicrobial activity despite having different proportions of carvacrol and thymol. The species evaluated in this study were *Lippia berlandieri* Schauer *Poliomintha longiflora*, with 33.78 and 18.35% relative concentrations of carvacrol, respectively, and 7.86% and 23.46% relative concentrations of thymol, respectively [111,116]. It was also observed that both species had higher antimicrobial activity against uropathogens *E. faecalis* and *E. coli* with MIC and CMB lower than 200 mg/L and 500–1000 mg/L, respectively. In contrast, *P. aeruginosa* was the uropathogen with the highest resistance, with a MIC/CMB higher than 2000 mg/L. These results suggest that Gram-negative bacteria are more resistant to terpenes than Gram-positive bacteria due to their outer membrane. In addition, they observed that the bactericidal effect of both oregano plants was similar, despite having a different proportion of thymol and carvacrol [111].

Comparative studies of different plant EOs have shown that oregano is one of the best plants with antimicrobial activity, mainly against uropathogens *E. coli* and *Enterococcus*. Likewise, studies carried out with the Greek oregano species (*O. vulgare*) show that this species also has antibacterial activity as the Mexican oregano species, analyzed by Zapién-Chavarría and collaborators [111]. These results are expected since Greek oregano has a higher concentration of Carvacrol (65.9–77.8%) than the Mexican oregano species [66,111,117]. However, comparing the antimicrobial activity of different oregano species would be interesting.

As mentioned above, studies have shown that *O. vulgare* EOs has higher antibacterial activity than other EOs, such as clove and thyme, against multidrug-resistant bacteria isolated from patients with UTIs, defining a MIC of 0.055 µL/mL for *O. vulgare* against *E. coli* [66]. Concerning recurrent UTIs, Xiao et al. [117] suggest that antibiotics currently used in the treatment of UTIs, such as nitrofurantoin, fosfomycin, sulfonamides, and quinolones, are mainly active when *E. coli* is in the growth phase, and therefore, uropathogens may be tolerant to antibiotics when they are metabolically quiescent. In this regard, Xiao and collaborates demonstrated that oregano EO was the best EO with the highest antibacterial activity compared with 139 other EOs. They established a MIC of 0.015% to inhibit the growth of *E. coli* and that at a concentration of 0.5% oregano EO can kill *E. coli* cells in the stationary phase. Even oregano EO showed superior antibacterial activity to tosufloxacin, an antibiotic characterized by high *E. coli* stationary phase activity. They also demonstrated that the combination of oregano EO with tosufloxacin, levofloxacin, or ciprofloxacin could be a strategy to eliminate *E. coli* cells in the stationary phase. In addition, the inhibitory ability of oregano against different uropathogens has been determined by measuring the inhibitory zone; for *E. coli*, an inhibitory zone of 29 mm was found, for *P. aeruginosa* of 27 mm, for *K. pneumoniae* of 20 mm, for *P. mirabilis* of 22 mm, for E. aerogenes of 21 mm, for *E. faecalis* of 21 mm, for *A. baumannii* of 22 mm, for *N. gonorrhoeae* of 24 mm, for *S. aureus* of 26 mm, and for *S. epidermis* of 20 mm [118]. Likewise, it has also been described that oregano oil at sub-inhibitory concentrations (MIC < 0.01%) was able to inhibit biofilm formation against the *E. coli* O6:H1 strain CFT073, which is a clinical isolate of a very virulent strain from the blood of a woman with acute pyelonephritis. This antibiofilm capacity is because carvacrol can prevent fimbriae production and reduce the swarming motility of bacteria. Besides, it has been associated that carvacrol can inhibit the hemagglutination ability and reduce the survival of bacteria in whole blood [8].

On the other hand, a study by Ebani et al. [17] evidence that in small animal practice, there is also the problem of multi-drug resistance, which is also related to the increased frequency of UTIs in these animals. In this interesting study Ebani and collaborates demonstrated that EO of *O. vulgare* possesses antimicrobial activity against multidrug-resistant strains of *E. coli*, *Enterococcus*, and antifungal activity on *C. albicans* and *C. famata* yeast, isolated from dogs and cats with severe cases of UTIs. In correlation with previous studies, Ebani and collaborators demonstrated that *O. vulgare* is one of the best plants with antimicrobial and antifungal effects compared to the other plants analyzed in their study star anise (*Illicium verum* Hook.f), basil (*Ocimum basilicum* L.), sage (*Salvia sclarea* L.) and thymus (*Thymus vulgaris* L). They determined that the MIC of *O. vulgare* against *E. coli* is 0.293–1.183 mg/mL, against *Enterococcus* is 1.183 mg/mL, against *C. albicans* is 0.09–3.6 mg/mL and against *C. famata* is 0.135–2.25 mg/mL. These results show that oregano has a significant effect against *E. coli*.

In another study conducted with EO from the species *O. glandulosum*, an endemic species from Africa-Mediterranean showed inhibitory activity against multidrug-resistant clinical strains of *K. pneumoniae* despite having a lower concentration of carvacrol (13%) than oregano Greek, determining a growth inhibitory activity of 43.5 +/− 6.7 mm and a MIC of 5.2 mg/mL. Additionally, through transmission electron microscopy, it was observed that *K. pneumoniae* cells under treatment with OE of O. *glandulosum* lose their typical rod shape and appear globular. At the same time, the protoplast shows large electron-dense areas, and the cell surface develops bumps and outer curly filaments [119].

In short, these studies show that different oregano species have antimicrobial and antifungal properties even though they have different proportions of carvacrol and thymol; this activity may be because these monoterpenes can block the synthesis of ergosterol, making the membrane porous and causing the death of yeast cells.

**Table 4:** Antimicrobial activity of oregano extracts.

Oregano Specie	Type of Extract	Phytochemicals	Uropathogen	MIC	MBC	Diameter of the Inhibition Zone (mm)	Ref.
*Lippia berlandieri* Schauer	Essential oil	Thymol (7.86%) and carvacrol (33.78%)	*E. faecalis and E. coli*	<200 mg/L	<200 mg/L	ND	[116]
*Poliomintha longiflora*	Essential oil	Thymol (23.46%) and carvacrol (18.35%)	*E. faecalis and E. coli*	<200 mg/L	<200 mg/L	ND	
*O. vulgare*	Essential oil	Carvacrol (68.96%)	*E. coli*	0.055 µL/mL	ND	24.5	[66]
			*K. pneumoniae*	ND	ND	22	
*O. vulgare*	Essential oil	Carvacrol (65.9%)	*E. coli*	0.293–1.183	ND	ND	[17]
			Enterococcus	1.183 mg/mL	ND	ND	
*O. vulgare*	Essential oil	Carvacrol (77.8%)	*E. coli* O6:H1 strain CFT073	0.01%	ND	ND	[8]
*O. vulgare*	Essential oil	Carvacrol (>50%)	*E. coli*	ND	ND	29	[118]
			*P. aeruginosa*	ND	ND	27	
			*K. pneumoniae*	ND	ND	20	
			*P. mirabilis*	ND	ND	22	
			*E. aerogenes*	ND	ND	21	
			*E. faecalis*	ND	ND	21	
			*A. baumannii*	ND	ND	22	
			*N. gonorrhoeae*	ND	ND	24	
			*S. aureus*	ND	ND	26	
			*S. epidermis*	ND	ND	20	
*O. vulgare*	Essential oil	ND	*E. coli* UTIs	0.015%	ND	ND	[117]
*O. glandulosum*	Essential oil	Thymol (33.2%), γ-terpinene (25.4%), *p*-cymene (16.1%), and carvacrol (13.0%)	K. *pneumoniae*	5.2 mg/mL	ND	43.5 ± 6.7	[119]

ND: Not determined, MIC: minimum inhibitory concentration, MBC: minimum bactericidal concentration.

## 7. Pepper

Pepper belongs to the Piperaceae family, which has more than 700 species. *Piper cubeba* is a native plant of Java and Borneo and is one of the most popular species. This species is generally found in Indonesia, India, medieval Europe, and North Africa. Economically, pepper is an essential source of its dried berries as they have several applications in perfumes, cosmetics, food preservatives, and therapeutic. Regarding medicinal properties, pepper herbal is used for treating many diseases since compounds have antioxidant, antibacterial, anti-inflammatory, and anticancer properties. The pepper is used mainly for digestive and respiratory disorders. Pepper species are rich in phytochemical compounds such as benzoic acids, amides, chromenos, terpenes, phenylpropanoids, lignans, alkaloids, fatty acids, and hydrocarbons. However, their biological activities are mainly attributed to compounds such as phenolic acids, flavonoids, and lignans such as cubebin, a bioactive compound with a wide range of biological activities such as antimicrobial, anticancer, and neuroprotective [106,120]. The antimicrobial effect of these plants’ extracts may be because EO can target the cell wall of bacterial cells; it can damage the proteins anchored to the cell wall, thus intervening in the formation and adhesion of biofilms. In comparison, the extracts attack and destroy the peptidoglycan leading to cell collapse. It has also been associated that these plants can inhibit oxidative stress, induce apoptosis and inhibit quorum sensing (QS) in pathogenic microbes [120].

Different toxicological studies have demonstrated the safety of different pepper species. For example, it was shown that *P. longum* L. species did not cause mortality in an acute (24 h) and chronic (90 days) model in mice. Similarly, it was shown that *P. betle* leaf extract was nontoxic to the glyoxalase system of Swiss albino mice after 2 weeks of oral administration at 1.5 and 10 mg/kg as well as the safety of the methanolic extract of *P. cubeba* fruit was also demonstrated up to a maximum dose of 2000 mg/kg body weight in female Wistar rats. Similar results were found in male Wistar rats with a dose range of 50 to 3000 mg/kg with *P. cubeba* EO [120]. The antimicrobial activity of pepper extracts is shown in Table 5.

The EO of *P. cubeba* is used for treating gonorrhea, dysentery, syphilis, abdominal pain, diarrhea, enteritis, and asthmatic diseases since it possesses antiparasitic, anti-inflammatory, and antimicrobial activities [120]. However, different studies have also found that pepper has antibacterial activity against several uropathogens. For instance, a recent study showed that aqueous-ethanolic extract (30/70) of *P. cubeba* at a concentration of 500 µg/mL possesses antibacterial activity against strains of *E. coli*, *S. saprophyticus*, *K. pneumonia*, and *P. mirabilis* finding a zone of inhibition by disk diffusion method of 18 ± 0.64, 19 ± 0.26, 21 ± 0.51, and 20 ± 0.41, respectively [106].

*P. cubeba* fruit extracted at a concentration of 50 mg/mL prepared with either acetone, methanol, or ethanol have been reported to have high to moderate antibacterial activity against multi-drug clinical isolated of *Klebsiella sp*., *S. aureus*, *E. coli*, *Enterococcus sp*., *Enterobacter sp*., and *P. aeruginosa*. The highest inhibition was found against *Enterococcus sp*. with a wide inhibition zone (17.6 ± 0.80 mm), followed by *E. coli* (16.3 ± 0.75 mm) and *P. aeruginosa* (15.3 ± 0.62 mm). It is also worth mentioning that *P. cubeba* extracts were more effective against Gram-positive bacteria; this may be because Gram-negative bacteria have a multilayered cell wall comprising of phospholipids and lipopolysaccharides that constitutes a barrier for the invasion of antimicrobial agents through the cell membrane [121].

Another pepper species that has been described for its activity against different types of antibacterial is *P. nigrum.* Alkaloid and phenolic compounds from this bell pepper species have been reported to have differential antibacterial activity against *S. aureus* and *E. coli* isolated from patients with UTIs. Phenolic compounds were found to have higher antibacterial activity against *S. aureus* and *E. coli*, with a zone of inhibition of 20.9 and 20.7 mm, respectively. In contrast, alkaloid compounds had a zone of inhibition of 10.5 mm for *S. aureus* and 18.4 mm for *E. coli* [122]. In the same way, similar results have been obtained with methanolic extracts of *P. nigrum* seed, showing that this extract has antimicrobial activity against strains isolated from patients with UTIs. For *E. faecalis,* an inhibition zone of 17 mm was observed; for *S. aureus,* 19 mm; for *Citrobacter freundii,* 17 mm; for *Enterobacter aerogenes,* 25 mm; for *K. pneumoniae,* 22 mm; and *P. mirabilis,* 18 mm. In addition, these methanolic extracts of *P. nigrum* seed were found to have better antimicrobial activity against *E. aerogenes* with MIC and MBC values of 1.51 mg/mL and 3.41 mg/mL, respectively [33]. However, in another study, controversial results were found with the methanolic extract of *P. nigrum* since it did not show antibacterial activity against *C. fruendi, K. pneumoniae, E. coli, P. aeruginosa, Proteus vulgaris, S. aureus,* and *E. aerogenes.* Possibly these contradictory results have to do with the fact that these strains were isolated from the urine of diabetic patients [123].

Likewise, aqueous and methanolic extracts of the fruit *P. longum* L. have been reported to have differential antimicrobial activity against multidrug-resistant strains isolated from patients with UTIs. Specifically, it has been reported that the aqueous extract of this plant has antibacterial activity against *P. aeruginosa* with a zone of inhibition of 12–18 mm, *S. aureus* with a zone of inhibition of 10–18 mm, and *E. coli* with a zone of inhibition of 13–22 mm. In contrast, *P. aeruginosa*, and *E. coli* were resistant to the methanolic extract. Additionally, the methanolic extract was found to be effective only against *S. aureus,* with a zone of inhibition of 8–14 mm and MIC of 3.75 mg/mL. These results show that it is necessary to use high concentrations to obtain the antimicrobial activity effects of crude extracts. Therefore, it is necessary to isolate the bioactive principle responsible for the antibacterial activity to use lower concentrations; however, further studies are needed to identify the compounds responsible for the bactericidal activity [124].

As mentioned previously, one of the reasons why uropathogens may be drug-resistant is due to their ability to form biofilms. However, it has also been reported that through the quorum sensing machinery, bacteria can coordinate their communication with each other and regulate the expression of their virulence genes. Therefore, anti-QS mechanisms are valid targets for developing new alternative agents to prevent biofilm formation and further infections [125,126]. In this regard, an interesting study focused on the synthesis *P. betle*-based synthesized silver nanoparticles (PbAgNPs) to evaluate their potential as anti-QS antibiofilm against *Serratia marcescens* (clinical isolate FJ584421 and ATCC 14756), and *P. mirabilis* (MTCC 425 and ATCC 7002). Overall, it was observed that PbAgNPs inhibited the expression of virulence-associated genes mediated by QS, such as prodigiosin and protease; also, these nanoparticles inhibited biofilm formation and swarming. Specifically, gene expression analysis showed that PbAgNPs at a concentration of 6 μg/mL downregulation virulence genes *fimA, fimC, flhD,* and *bsmB* in *S. marcescens* and *flhB, flhD,* and *rsbA* in *P. mirabilis*. Moreover, the MIC of the PbAgNPs was 16 μg/mL for *S. marcescens* strains and 32 μg/mL for *P. mirabilis* strains [127].

In conclusion, these studies demonstrate that aqueous, methanolic, and ethanolic extracts of pepper have differential antibacterial activity against bacteria. Furthermore, although there are several studies on the use of pepper as an antibacterial agent, studies need to specify which part of the plant was processed and identify the compounds with antibacterial activity and their proportion.

**Table 5:** Antimicrobial activity of pepper extract.

Pepper Specie	Type of Extract	Phytochemicals	Uropathogen	MIC	MBC	Diameter of the Inhibition Zone (mm)	Ref.
*P. cubeba*	Acetone extract	Flavonoids, steroids, tannins, reducing sugars, and triterpenoids	*Enterococcus sp.*	ND	ND	15.2 ± 0.52	[121]
			*P. aeruginosa*	ND	ND	15.3 ± 0.62	
			*E. coli*	ND	ND	16.3 ± 0.75	
	Methanolic extract	Saponins, flavonoids, steroids, tannins, reducing sugars, cardiac glycosides, and triterpenoids	*Enterococcus sp.*	ND	ND	17.6 ± 0.80	
			*P. aeruginosa*	ND	ND	13.2 ± 0.06	
			*E. coli*	ND	ND	15.0 ± 0.30	
	Ethanolic extract	Flavonoids, steroids, tannins, reducing sugars, cardiac glycosides, and triterpenoids	*Enterococcus sp.*	ND	ND	11.3 ± 0.16	
			*P. aeruginosa*	ND	ND	9.6 ± 0.34	
			*E. coli*	ND	ND	8.5 ± 0.17	
*P. cubeba*	Aqueous-ethanolic (30/70) extract	Flavonoids, alkaloids, sterols, phenols, and tannins	*E. coli*	ND	ND	18 ± 0.64	[106]
			*S. saprophyticus*	ND	ND	19 ± 0.26	
			*K. pneumoni*	ND	ND	21 ± 0.51	
			*P. mirabilis*	ND	ND	20 ± 0.41	
*P.longum*	Aqueous extract	Alkaloids, flavonoids, triterpenes, tannins, coumarins, cardiac glycosides, anthraquinones, glycosides, saponins	*P. aeruginosa*	ND	ND	19 ± 0.26	[124]
			*S. aureus*	ND	ND	21 ± 0.51	
			*E. coli*	ND	ND	20 ± 0.41	
	Methanolic extract	Alkaloids, flavonoids, triterpenes, tannins, coumarins, cardiac glycosides, anthraquinones, glycosides, saponins	*P. aeruginosa*	1.875 mg/mL	ND	ND	
			*S. aureus*	3.75 mg/mL	ND	8–14	
			*E. coli*	*0.937* mg/mL	ND	ND	
*P. betle*	Aqueous extract	ND	*S. marcescens*	16 μg/mL	ND	ND	[127]
			*P.mirabilis*	32 μg/mL	ND	ND	
*P. nigrum*	Methanolic extract	Capsaicin and 2- dihidrocapsaicin	*S. aureus*	ND	ND	10.5	[122]
			*E. coli*	ND	ND	18.4	
	Ethanolic extract	Gallic acid, *trans*-p-feruloyl-b-D-glucopyranoside, *trans*-p-sinapyl-b-D-glucopyranoside, quercetin 3-O-R-L-rhamnopyranoside-7-O-a-D-glucopyranosyl, quercetin 3-O-R-L-rhamnopyranoside, luteolin 6-C-a-D-glucopyranoside-8-C-R-L-arabinopyranoside, luteolin 7-O-[2-(b-D-apiofuranosyl)-b-D-glucopyranoside-8-C-R-L-arabinopyranoside, luteolin 7-O-[2-(b-D-apiofuranosyl)-4-(b-D-glucopyranosyl), kaempferol and coumarins	*S. aureus*	ND	ND	20.9	
			*E. coli*	ND	ND	20.7	
*P. nigrum*	Methanolic extract	Glycosides, terpenoids, carbohydrates, tannins and steroids	*E. faecalis*	9.63 mg/mL	4.27 mg/mL	17	[33]
			*S. aureus*	21.67 mg/mL	9.63 mg/mL	19	
			*C freundii*	21.67 mg/mL	9.63 mg/mL	17	
			*E aerogenes*	3.41 mg/mL	1.51 mg/mL	25	
			*K pneumoniae*	4.27 mg/mL	3.41 mg/mL	22	
			*P. mirabilis*	9.63 mg/mL	4.27 mg/mL	18	

ND: not determined; MIC: minimum inhibitory concentration; MBC: minimum bactericidal concentration.

## 8. Rosemary

The evergreen plant of rosemary belongs to the Lamiaceae family. It is native to the Caucasus and the Eastern Mediterranean, although had been introduced into many regions of France, Italy, China, the United States, and Mexico [128,129]. Fresh and dried rosemary leaves are widely used as a spice in soups, stews, fish, bread, stuffings, meats, and roasted vegetables. Rosemary is also a natural preservative in the food industry, with an adequate daily intake (ADI) of 0–0.3 mg/kg body weight for rosemary extract, expressed as carnosic acid plus carnosol [130]. Furthermore, rosemary is a medicinal plant whit antioxidant, antihyperglycemic, anti-inflammatory, antiviral and antimicrobial properties [128,131,132].

The extract of rosemary can be obtained from roots, stems, leaves, flowers, fruits, seeds, and bark through maceration, hydrodistillation, Soxhlet extraction, microwave or ultrasound-assisted extraction, accelerated solvent extraction, and supercritical fluid extraction, using solvents such as ethanol, methanol, acetone, hexane, and water [131,132]. However, rosemary leaves extracts are the most common extracts used. The bioactive compounds isolated from the leaves of rosemary EOs or non-water extracts are borneol, caffeic acid, camphor, carnosic acid, carnosol, chlorogenic acid, 1–8-cineole, eucalyptol, eugenol, luteolin, α and β-pinene, rosmadial, rosmanol, rosmarinic acid, rosmaquinones A and B, secohinokio and ursolic acid. The water extract of rosemary only contains rosmarinic acid [128,131,133].

The antimicrobial activity of rosemary is due to caffeic acid, camphor, carnosic acid, carnosol, 1,8-cineole, borneol, epirosmanol, isorosmanol, luteolin, rosmaridiphenol, rosmarinic acid, and rosmanol. These bioactive compounds synergize and could interact with the cell membrane, thus altering the transport of nutrients and ions that produce a loss of membrane structure and functionality [133,134]. In addition, it described that 1,8-cineole cause membrane disintegration and nucleoplasm reduction; these effects are similar to the action of nisin, an antibiotic used as a food additive, which causes cytoplasmic leakage [135]. The antimicrobial activity of rosemary leaves extracts is presented in Table 6.

Some pathogens, such as *E. coli*, can form biofilms and lead to recurrent UTIs. Rosemary EO could inhibit 86.36% of the biofilm activity of *E. coli* isolated strain of patients aged 2 months to 90 years and MIC and MCB values to 1.56–3.125 and 12.5 mg/mL, respectively [136]. In addition, *E. coli* strains collected from prostatitis patients have been reported to have increased biofilm production compared to *E. coli* strains that cause cystitis and pyelonephritis. Moreover, the use of rosemary EO has a MIC value of 10 mg/mL for strong and moderate *E. coli* biofilm producers and showed a synergistic effect whit gentamicin and ciprofloxacin against both *E. coli* biofilms [137]. In addition, a synergism with ceftazidime in *E. coli* isolated strains from urinary samples of patients with UTIs [138]. The methanolic extract of rosemary also inhibits the biofilm formation of *E. coli* strains isolated from patients with UTIs in a concentration-dependent manner and has MIC and MCB values of 5 and 10 mg/mL, respectively [139].

In addition, the antimicrobial activity of methanolic rosemary extract was tested in isolated *E. coli* obtained from urinary samples of patients. The inhibition zone and MIC of rosemary extract were 5.4 mm and 64 µg/mL, respectively. When used with ceftazidime or ceftriaxone, it increases the inhibition zone and thus the sensitivity of *E. coli*; this synergism effect with antibiotics should be tested because when rosemary is added with gentamicin, ciprofloxacin and trimethoprim had no synergistic effects [138]. On the other hand, the antimicrobial activity of the ethanolic rosemary extract was tested on isolated Gram-negative and Gram-positive uropathogens. The results indicate that at the highest concentration tested (400 µg/mL) of this extract, it had no antimicrobial activity against Gram-negative strains.

In contrast, Gram-positive uropathogens have MIC values of 70–130 µg/mL for *S. saprophyticus*, *S. epidermidis*, and *E. faecalis* and MCB values of 130, >400, and 300 µg/mL, respectively. However, when the ethanolic extracts of rosemary are fractioned with different solvents, the different fractions obtained have antimicrobial activity against Gram-negative uropathogens such as *P. aeuruginosa*, and *P.mirabilis*. The MIC and MCB values increase or decrease for Gram-positive isolated strains, which depend on the isolated fraction containing different bioactive compounds [140].

As mentioned above, the inhibitory effect of rosemary extract in *E. coli* strains isolated from urine is widely demonstrated. Nevertheless, this pathogen could be distributed in the abdominal cavity, bronchia, wounds, and blood; therefore, rosemary extracts could inhibit the growth of *E. coli* strains regardless of their location and with a different pattern of resistance [141]. Moreover, EO rosemary has an antibacterial effect against other pathogens, such as *S. aureus*, *K. pneumoniae,* and *P. vulgaris* strains, with a MIC of around 0.06 to 0.16 mg/mL. However, these values are higher than the ciprofloxacin and nitrofurantoin (0.16–0.2 and 16–32 µg/mL, respectively); some strains resist antibiotics, such as *P. vulgaris* strain to nitrofurantoin [142]. Therefore, rosemary extract could treat UTIs and solve antibiotic resistance.

Finally, rosemary leaves, century herbs, and lovage roots are commercialized in an herbal medicinal product known as Canephron^®^ N (Bionorica, SE, Germany), which exerts antimicrobial properties, among others. It is described that this herbal medicine can reduce the rate of UTIs recurrence and may decrease the side effects of antibiotics, such as diarrhea and abdominal pain [143,144,145].

**Table 6:** Antimicrobial activity of rosemary extracts from leaves.

Type of Extract	Phytochemicals	Uropathogen	MIC	MBC	Diameter of the Inhibition Zone (mm)	Ref.
Ethanolic extract	Rosmarinic acid, rosmanol, geniposide	*S. saprophyticus*	130 mg/mL	130 mg/mL	ND	[140]
		*S. epidermidis*	70 mg/mL	>400 mg/mL	ND	
		*E. faecalis*	100 mg/mL	300 mg/mL	ND	
Essential oil	1,8-cineole (46.4%), camphor (11.4%), a-pinene (11%), b-pinene (9.2%), camphene (5.02%)	*E. coli*	18.25–19.75 mL/mL	ND	ND	[73]
Essential oil	1,8-cineole, camphor, a-pinene, b-pinene, camphene	*E. coli*	10 mg/mL	ND	ND	[137]
Essential oil	1,8-cineole (17.16%), a-pinene (16.95%), verbenone (15.78%), camphor (8.08%)	*S. aureus*	0.06–0.16 mg/mL	0.06–0.16 mg/mL	7–9.6	[142]
		*K. pneumoniae*	0.06–0.16 mg/mL	0.06–0.16 mg/mL	7–9.6	
		*P. vulgaris*	0.06–0.16 mg/mL	0.06–0.16 mg/mL	7–9.6	
Methanolic extract	Caffeic acid, borneol, limonene, camphor	*E. coli*	64 mg/mL	ND	5.48	[138]
Methanolic extract	Caffeic acid, borneol, limonene, camphor	*E. coli*	5 mg/mL	10 mg/mL	ND	[139]

ND: not determined; MIC: minimum inhibitory concentration; MBC: minimum bactericidal concentration.

## 9. Concluding Remarks and Future Directions

UTIs are the most common bacterial infections with high worldwide prevalence and are usually treated with various antibiotics. However, some strains of uropathogenic microorganisms develop antibiotic resistance and form biofilms that impede the host’s immune response and antimicrobial treatment. Therefore, plant extracts have been tested in animal models and clinically isolated uropathogens as an alternative or complementary treatment. 

Several methods are used to extract plants’ bioactive compounds from fruits, leaves, seeds, and bark. The extraction method and solvent used will determine the chemical composition of plant-based extracts, which contain various compounds such as flavonoids, tannins, lignans, alkaloids, saponins, terpenoids, anthraquinones, and glycosides which exert antimicrobial properties. Nevertheless, some of these compounds have been identified as the main ones responsible for antimicrobial activity, such as trans-cinnamaldehyde, eugenol, carvacrol, and 1–8 cineole in cinnamon, clove, oregano, and rosemary, respectively. The main mechanisms of action of these phytochemicals are loss of membrane structure and functionality, depletion of ATP intracellular levels, and reduction of key enzymes of the citric acid cycle pathway. Nevertheless, all the components in spice EOs also have antimicrobial activity and synergistic effects with other compounds present in these extracts. However, the mechanisms could better describe and must be elucidated to be combined with antibiotics or other compounds and have a greater efficacy or a synergistic effect.

Although spices are considered safe for consumption, and their EOs’ antimicrobial efficacy has been widely demonstrated, they are not ready to be used as an alternative or complementary treatment for UTIs in patients because of the lack of studies about the bioavailability, distribution, and excretion of the phytochemicals on EO, so it should be studied and improved in order to enhance the antimicrobial activity. The research should be focused on demonstrating the above because they are a promising treatment that could solve the antibiotic resistance problem and biofilm formation in tissues or inert surfaces.

## Figures and Tables

**Table 3:** Antimicrobial activity of *Cuminum cyminum* extracts.

Type of Extract	Phytochemicals	Uropathogen	MIC	MBC	Diameter of the Inhibition Zone (mm)	Ref.
Essential oil	Cuminaldehyde, α-thujene, α,b-pinene, *p*-cymene, g-terpinene, cumin oils	*K. pneumoniae*	0.8–3.5 μg/mL	ND	ND	[103]
Essential oil	Cuminaldehyde, α-thujene, α,b-pinene, *p*-cymene, g-terpinene, cumin oils	*E. coli*	10–50 ppm and 100–250 ppm *	ND	ND	[102]
Essential oil	Cuminaldehyde, α-thujene, α,b-pinene, *p*-cymene, g-terpinene, cumin oils	*E. coli*	0.25 mg/mL	0.5 mg/mL	23	[104]
		*K. pneumoniae*	0.25 mg/mL	0.5 mg/mL	22	
		*p. aeruginosa*	0.25 mg/mL	0.5 mg/mL	20	
		*S. agalactiae*	0.25 mg/mL	0.5 mg/mL	21	
		group A *streptococci*	0.015 mg/mL	0.03 mg/mL	20	
		*E. faecalis*	0.125 mg/mL	0.25 mg/mL	20	
		*S.epidermidis*	0.25 mg/mL	0.5 mg/mL	10	
		*S. aureus*	ND	ND	7	
		*S. saprophyticus*	0.25 mg/mL	0.5 mg/mL	20	
Essential oil	Cuminaldehyde, α-thujene, α,b-pinene, *p*-cymene, g-terpinene, cumin oils	*S. aureus*	1161 μg/mL	ND	45	[81]
		*P. aeruginosa*	84.97 μg/mL	ND	8	
		*K. pneumoniae*	204.87 μg/mL	ND	12	
		*E. coli*	7.219 μg/mL	ND	52	
Ethanolic extract	ND	*E. coli*	0.125 mg/mL	0.25 mg/mL	22	[104]
		*K. pneumoniae*	0.125 mg/mL	0.25 mg/mL	22	
		*P. aeruginosa*	0.25 mg/mL	0.5 mg/mL	20	
		*S. agalactiae*	ND	ND	7	
		group A *streptococci*	0.125 mg/mL	0.25 mg/mL	23	
		*E. faecalis*	0.125 mg/mL	0.25 mg/mL	23	
		*S. epidermidis*	0.125 mg/mL	0.25 mg/mL	25	
		*S. aureus*	0.125 mg/mL	0.25 mg/mL	20	
		*S. saprophyticus*	0.25 mg/mL	0.5 mg/mL	23	
Aqueous-ethanolic (30/70) extract	Carbohydrates, flavonoids, protein, alkaloids, phenols	*E. coli*	ND	ND	26	[106]
		*K. pneumonia*	ND	ND	22	
		*S. saprophyticus*	ND	ND	25	
		*P. mirabilis*	ND	ND	21.5	

* Depending on the isolated strain; ND: not determined; MIC: minimum inhibitory concentration; MBC: minimum bactericidal concentration.

## References

[1] Yang X., Chen H., Zheng Y., Qu S., Wang H., Yi F. (2022). Disease burden and long-term trends of urinary tract infections: A worldwide report. Front. Public Health.

[2] Ballesteros-Monrreal M.G., Arenas-Hernández M.M.P., Barrios-Villa E., Juarez J., Álvarez-Ainza M.L., Taboada P., De la Rosa-López R., Bolado-Martínez E., Valencia D. (2021). Bacterial Morphotypes as Important Trait for Uropathogenic E. coli Diagnostic; a Virulence-Phenotype-Phylogeny Study. Microorganisms.

[3] Bien J., Sokolova O., Bozko P. (2012). Role of Uropathogenic *Escherichia coli* Virulence Factors in Development of Urinary Tract Infection and Kidney Damage. Int. J. Nephrol..

[4] Asadi Karam M.R., Habibi M., Bouzari S. (2019). Urinary tract infection: Pathogenicity, antibiotic resistance and development of effective vaccines against Uropathogenic *Escherichia coli*. Mol. Immunol..

[5] Naseer F. (2018). Antibacterial Activity of Medicinal Plants (Clove, Cinnamon, Garlic) Extracts and their Combined Effect with Antibiotics in Urinary Tract Infection Caused by *Escherichia coli*. Int. J. Pharm. Pharmacol..

[6] Kim A., Ahn J.H., Choi W.S., Park H.K., Kim S., Paick S.H., Kim H.G. (2021). What is the Cause of Recurrent Urinary Tract Infection? Contemporary Microscopic Concepts of Pathophysiology. Int. Neurourol. J..

[7] Murray B.O., Flores C., Williams C., Flusberg D.A., Marr E.E., Kwiatkowska K.M., Charest J.L., Isenberg B.C., Rohn J.L. (2021). Recurrent Urinary Tract Infection: A Mystery in Search of Better Model Systems. Front. Cell. Infect. Microbiol..

[8] Lee J.-H., Kim Y.-G., Lee J. (2017). Carvacrol-rich oregano oil and thymol-rich thyme red oil inhibit biofilm formation and the virulence of uropathogenic *Escherichia coli*. J. Appl. Microbiol..

[9] Lucas M., Macías J., Cañarte J. (2021). Perfil de sensibilidad a antimicrobianos como principal criterio para la selección del tratamiento de infecciones del tracto urinario. Revisión Sistemática. Rev. Kasmera.

[10] Flores-Mireles A.L., Walker J.N., Caparon M., Hultgren S.J. (2015). Urinary tract infections: Epidemiology, mechanisms of infection and treatment options. Nat. Rev. Microbiol..

[11] Kaur R., Kaur R. (2021). Symptoms, risk factors, diagnosis and treatment of urinary tract infections. Postgrad. Med. J..

[12] Álvarez-Martínez F.J., Barrajón-Catalán E., Herranz-López M., Micol V. (2021). Antibacterial plant compounds, extracts and essential oils: An updated review on their effects and putative mechanisms of action. Phytomedicine.

[13] Christaki S., Moschakis T., Kyriakoudi A., Biliaderis C.G., Mourtzinos I. (2021). Recent advances in plant essential oils and extracts: Delivery systems and potential uses as preservatives and antioxidants in cheese. Trends Food Sci. Technol..

[14] Altemimi A., Lakhssassi N., Baharlouei A., Watson D., Lightfoot D. (2017). Phytochemicals: Extraction, Isolation, and Identification of Bioactive Compounds from Plant Extracts. Plants.

[15] Heinrich M., Jalil B., Abdel-Tawab M., Echeverria J., Kulić Ž., McGaw L.J., Pezzuto J.M., Potterat O., Wang J.-B. (2022). Best Practice in the chemical characterisation of extracts used in pharmacological and toxicological research—The ConPhyMP—Guidelines12. Front. Pharmacol..

[16] Sakkas H., Papadopoulou C. (2017). Antimicrobial Activity of Basil, Oregano, and Thyme Essential Oils. J. Microbiol. Biotechnol..

[17] Ebani V., Nardoni S., Bertelloni F., Pistelli L., Mancianti F. (2018). Antimicrobial Activity of Five Essential Oils against Bacteria and Fungi Responsible for Urinary Tract Infections. Molecules.

[18] FDA CPG Sec 525.750 Spices—Definitions. https://www.fda.gov/regulatory-information/search-fda-guidance-documents/cpg-sec-525750-spices-definitions.

[19] Vázquez-Fresno R., Rosana A.R.R., Sajed T., Onookome-Okome T., Wishart N.A., Wishart D.S. (2019). Herbs and Spices- Biomarkers of Intake Based on Human Intervention Studies—A Systematic Review. Genes Nutr..

[20] Peter K.V., Shylaja M.R. (2012). Introduction to herbs and spices: Definitions, trade and applications. Handbook of Herbs and Spices.

[21] De La Torre J.E., Gassara F., Kouassi A.P., Brar S.K., Belkacemi K. (2017). Spice use in food: Properties and benefits. Crit. Rev. Food Sci. Nutr..

[22] Mercado-Mercado G., Carrillo L.d.l.R., Wall-Medrano A., Díaz J.A.L., Álvarez-Parrilla E. (2013). Compuestos polifenólicos y capacidad antioxidante de especias típicas consumidas en México. Nutr. Hosp..

[23] OEC Spices. https://oec.world/es/profile/hs/spices.

[24] Sobel J. (2002). Investigation of Multistate Foodborne Disease Outbreaks. Public Health Rep..

[25] Bi X., Lim J., Henry C.J. (2017). Spices in the management of diabetes mellitus. Food Chem..

[26] Rastogi S., Pandey M.M., Kumar Singh Rawat A. (2017). Spices: Therapeutic Potential in Cardiovascular Health. Curr. Pharm. Des..

[27] Rubió L., Motilva M.-J., Romero M.-P. (2013). Recent Advances in Biologically Active Compounds in Herbs and Spices: A Review of the Most Effective Antioxidant and Anti-Inflammatory Active Principles. Crit. Rev. Food Sci. Nutr..

[28] Satheeshkumar N., Vijayan R.S.K., Lingesh A., Santhikumar S., Vishnuvardhan C., Essa M., Akbar M., Guillemin G. (2016). Spices: Potential therapeutics for Alzheimer’s disease. The Benefits of Natural Products for Neurodegenerative Diseases.

[29] Anderson C.A., Cobb L.K., Miller E.R., Woodward M., Hottenstein A., Chang A.R., Mongraw-Chaffin M., White K., Charleston J., Tanaka T. (2015). Effects of a behavioral intervention that emphasizes spices and herbs on adherence to recommended sodium intake: Results of the SPICE randomized clinical trial. Am. J. Clin. Nutr..

[30] Cowan M.M. (1999). Plant Products as Antimicrobial Agents. Clin. Microbiol. Rev..

[31] Calucci L., Pinzino C., Zandomeneghi M., Capocchi A., Ghiringhelli S., Saviozzi F., Tozzi S., Galleschi L. (2003). Effects of γ-Irradiation on the Free Radical and Antioxidant Contents in Nine Aromatic Herbs and Spices. J. Agric. Food Chem..

[32] Mickymaray S., Al Aboody M.S. (2019). In Vitro Antioxidant and Bactericidal Efficacy of 15 Common Spices: Novel Therapeutics for Urinary Tract Infections?. Medicina.

[33] Rath S., Padhy R.N. (2014). Monitoring in vitro antibacterial efficacy of 26 Indian spices against multidrug resistant urinary tract infecting bacteria. Integr. Med. Res..

[34] Opara E., Chohan M. (2014). Culinary Herbs and Spices: Their Bioactive Properties, the Contribution of Polyphenols and the Challenges in Deducing Their True Health Benefits. Int. J. Mol. Sci..

[35] Burt S. (2004). Essential oils: Their antibacterial properties and potential applications in foods—A review. Int. J. Food Microbiol..

[36] Bashir R., Naeem N., Waheed A., Sultan N. (2021). Advantageous Impact of Spices in Controlling Urinary Tract Infections. Eur. J. Med. Health Sci..

[37] Cadena B.R. Cocina Mexicana: Las Especias Más Usadas. https://aprende.com/blog/gastronomia/comida-mexicana/especias-cocina-mexicana/.

[38] Iturriaga J. La Cocina Mexicana: Patrimonio Cultural de la Humanidad. https://go.gale.com/ps/i.do?id=GALE%7CA296952025&sid=googleScholar&v=2.1&it=r&linkaccess=abs&issn=14023357&p=IFME&sw=w&userGroupName=anon~fd3aadd5.

[39] Nabavi S., Di Lorenzo A., Izadi M., Sobarzo-Sánchez E., Daglia M., Nabavi S. (2015). Antibacterial Effects of Cinnamon: From Farm to Food, Cosmetic and Pharmaceutical Industries. Nutrients.

[40] Vasconcelos N.G., Croda J., Simionatto S. (2018). Antibacterial mechanisms of cinnamon and its constituents: A review. Microb. Pathog..

[41] Rasool N., Saeed Z., Pervaiz M., Ali F., Younas U., Bashir R., Bukhari S.M., Mahmood Khan R.R., Jelani S., Sikandar R. (2022). Evaluation of essential oil extracted from ginger, cinnamon and lemon for therapeutic and biological activities. Biocatal. Agric. Biotechnol..

[42] Mbaveng A.T., Kuete V. (2017). Cinnamon species. Medicinal Spices and Vegetables from Africa.

[43] Rao P.V., Gan S.H. (2014). Cinnamon: A Multifaceted Medicinal Plant. Evid. -Based Complement. Altern. Med..

[44] Błaszczyk N., Rosiak A., Kałużna-Czaplińska J. (2021). The Potential Role of Cinnamon in Human Health. Forests.

[45] Kowalska J., Tyburski J., Matysiak K., Jakubowska M., Łukaszyk J., Krzymińska J. (2021). Cinnamon as a Useful Preventive Substance for the Care of Human and Plant Health. Molecules.

[46] Jayaprakasha G.K., Rao L.J.M. (2011). Chemistry, Biogenesis, and Biological Activities of Cinnamomum zeylanicum. Crit. Rev. Food Sci. Nutr..

[47] Hussain S., Rahman R., Mushtaq A., Zerey-Belaskri A. (2017). El Clove: A review of a precious species with multiple uses. Int. J. Chem. Biochem. Sci..

[48] Yun J.-W., You J.-R., Kim Y.-S., Kim S.-H., Cho E.-Y., Yoon J.-H., Kwon E., Jang J.-J., Park J.-S., Kim H.-C. (2018). In vitro and in vivo safety studies of cinnamon extract (Cinnamomum cassia) on general and genetic toxicology. Regul. Toxicol. Pharmacol..

[49] Gu D.-T., Tung T.-H., Jiesisibieke Z.L., Chien C.-W., Liu W.-Y. (2022). Safety of Cinnamon: An Umbrella Review of Meta-Analyses and Systematic Reviews of Randomized Clinical Trials. Front. Pharmacol..

[50] Modi P.I., Parikh J.K., Desai M.A. (2019). Sonohydrodistillation: Innovative approach for isolation of essential oil from the bark of cinnamon. Ind. Crops Prod..

[51] Kumar V., Marković T., Emerald M., Dey A. (2016). Herbs: Composition and dietary importance. Encyclopedia of Food and Health.

[52] Pereira W.A., Pereira C.D.S., Assunção R.G., da Silva I.S.C., Rego F.S., Alves L.S.R., Santos J.S., Nogueira F.J.R., Zagmignan A., Thomsen T.T. (2021). New Insights into the Antimicrobial Action of Cinnamaldehyde towards *Escherichia coli* and Its Effects on Intestinal Colonization of Mice. Biomolecules.

[53] Waty S., Suryanto D. (2018). Yurnaliza Antibacterial activity of cinnamon ethanol extract (cinnamomum burmannii) and its application as a mouthwash to inhibit streptococcus growth. IOP Conf. Ser. Earth Environ. Sci..

[54] Zhang Y., Liu X., Wang Y., Jiang P., Quek S. (2016). Antibacterial activity and mechanism of cinnamon essential oil against *Escherichia coli* and Staphylococcus aureus. Food Control.

[55] Thiyagarajan S., John S. (2020). Antimicrobial activity of Cinnamomum zeylanicum aqueous extract against bacteria and fungi responsible for urinary tract infection. Int. J. Health Allied Sci..

[56] Utchariyakiat I., Surassmo S., Jaturanpinyo M., Khuntayaporn P., Chomnawang M.T. (2016). Efficacy of cinnamon bark oil and cinnamaldehyde on anti-multidrug resistant Pseudomonas aeruginosa and the synergistic effects in combination with other antimicrobial agents. BMC Complement. Altern. Med..

[57] Zenati F., Benbelaid F., Khadir A., Bellahsene C., Bendahou M. (2014). Antimicrobial effects of three essential oils on multidrug resistant bacteria responsible for urinary infections. J. Appl. Pharm. Sci..

[58] Dhore M.R., Jha A.R. (2019). Antimicrobial activity of Allium cepa and Cinnamomum zeylanicum against common bacteria causing urinary tract infections: In vitro study. Int. J. Basic Clin. Pharmacol..

[59] Narayanan A., Muyyarikkandy M.S., Mooyottu S., Venkitanarayanan K., Amalaradjou M.A.R. (2017). Oral supplementation of trans -cinnamaldehyde reduces uropathogenic *Escherichia coli* colonization in a mouse model. Lett. Appl. Microbiol..

[60] Rafeeq H., Sharba Z. (2022). Study the Effect of Cinnamon and Tea Tree Oils on Biofilm Formation of Klebsiella Pneumoniae. J. Appl. Sci. Nanotechnol..

[61] Idris F.Z., Habibu U.A. (2021). In-vitro antibacterial activity of cinnamon bark extracts on clinical multi-drug resistant (mdr) Staphylococcus aureus, Klebsiella pneumoniae and Pseudomonas aeruginosa isolates. Bayero J. Pure Appl. Sci..

[62] Cock E., Cheesman M., Goyal M.R. (2018). Plants of the genus syzygium (Myrtaceae): A review on ethnobotany, medicinal properties, and phytochemistry. Bioactive Compounds of Medicinal Plants.

[63] Ayushi, Khan U.A., Danish S.M., Mohammad, Parveen U. (2020). A review on biological and therapeutic uses of Syzygium aromaticum Linn. (Clove): Based on phyto-chemistry and pharmacological evidences. Int. J. Bot. Stud..

[64] Shan B., Cai Y.Z., Sun M., Corke H. (2005). Antioxidant Capacity of 26 Spice Extracts and Characterization of Their Phenolic Constituents. J. Agric. Food Chem..

[65] National Center for Biotechnology Information PubChem Compound Summary for CID 3314, Eugenol. https://pubchem.ncbi.nlm.nih.gov/compound/Eugenol.

[66] Băicuș A., Mattuzzi F.C., Paraschiv A.M., Dinu R.-S., Dumitrescu M.C., Marinescu A.A., Ionescu D., Dragos D. (2022). Antibacterial Activity of Clove, Oregano, Thyme, Eucalyptus, and Tea Tree Essential Oils against *Escherichia coli* and Klebsiella pneumoniae strains. Rev. Rom. Med. Lab..

[67] Cui H., Zhao C., Lin L. (2015). The specific antibacterial activity of liposome-encapsulated Clove oil and its application in tofu. Food Control.

[68] Faujdar S., Bisht D., Sharma A. (2020). Antibacterial activity of Syzygium aromaticum (clove) against uropathogens producing ESBL, MBL, and AmpC beta-lactamase: Are we close to getting a new antibacterial agent?. J. Fam. Med. Prim. Care.

[69] Mytle N., Anderson G.L., Doyle M.P., Smith M.A. (2006). Antimicrobial activity of clove (Syzgium aromaticum) oil in inhibiting Listeria monocytogenes on chicken frankfurters. Food Control.

[70] Nuñez L., D’Aquino M. (2012). Microbicide activity of clove essential oil (Eugenia caryophyllata). Braz. J. Microbiol..

[71] Guan W., Li S., Yan R., Tang S., Quan C. (2007). Comparison of essential oils of clove buds extracted with supercritical carbon dioxide and other three traditional extraction methods. Food Chem..

[72] Hatami T., Johner J.C.F., Zabot G.L., Meireles M.A.A. (2019). Supercritical fluid extraction assisted by cold pressing from clove buds: Extraction performance, volatile oil composition, and economic evaluation. J. Supercrit. Fluids.

[73] Dąbrowski M., Sienkiewicz M., Zielińska-Bliźniewska H., Dąbrowska M., Seredyńska M., Kochan E. (2018). Antibiotic resistance among *Escherichia coli* urinary isolates and their susceptibility to clove essential oil. Ann. Univ. Mariae Curie-Sklodowska Sect. C Biol..

[74] Raut J.S., Karuppayil S.M. (2014). A status review on the medicinal properties of essential oils. Ind. Crops Prod..

[75] Roy S., Chaurvedi P., Chowdhary A. (2015). Evaluation of antiviral activity of essential oil of Trachyspermum Ammi against Japanese encephalitis virus. Pharmacogn. Res..

[76] Swamy M.K., Akhtar M.S., Sinniah U.R. (2016). Antimicrobial Properties of Plant Essential Oils against Human Pathogens and Their Mode of Action: An Updated Review. Evidence-Based Complement. Altern. Med..

[77] Goñi P., López P., Sánchez C., Gómez-Lus R., Becerril R., Nerín C. (2009). Antimicrobial activity in the vapour phase of a combination of cinnamon and clove essential oils. Food Chem..

[78] Devi K.P., Nisha S.A., Sakthivel R., Pandian S.K. (2010). Eugenol (an essential oil of clove) acts as an antibacterial agent against Salmonella typhi by disrupting the cellular membrane. J. Ethnopharmacol..

[79] Rhayour K., Bouchikhi T., Tantaoui-Elaraki A., Sendide K., Remmal A. (2003). The Mechanism of Bactericidal Action of Oregano and Clove Essential Oils and of Their Phenolic Major Components on *Escherichia coli* and Bacillus subtilis. J. Essent. Oil Res..

[80] Valle D.L., Cabrera E.C., Puzon J.J.M., Rivera W.L. (2016). Antimicrobial Activities of Methanol, Ethanol and Supercritical CO2 Extracts of Philippine Piper betle L. on Clinical Isolates of Gram Positive and Gram Negative Bacteria with Transferable Multiple Drug Resistance. PLoS ONE.

[81] Mahmoud A.M., El-Baky R.M.A., Ahmed A.B.F., Fadl G. (2016). Antibacterial Activity of Essential Oils and in Combination with Some Standard Antimicrobials against Different Pathogens Isolated from Some ClinicalSpecimens. Am. J. Microbiol. Res..

[82] Rakshit M., Ramalingam C. (2010). Screening and Comparision of Antibacterial Activity of Indian Spices. J. Exp. Sci..

[83] Rosarior V.L., Lim P.S., Wong W.K., Yue C.S., Yam H.C., Tan S.-A. (2021). Antioxidant-rich Clove Extract, A Strong Antimicrobial Agent against Urinary Tract Infections-causing Bacteria in vitro. Trop. Life Sci. Res..

[84] Code of Federal Regulations (2015). Chapter I. Food and Drug Administration. Subchapter E. Animal drugs, feeds, and related products. Food and Drugs.

[85] Shalaby S., El-Din M., Abo-Donia S., Mettwally M., Attia Z. (2011). Toxicological effects of essential oils from Eucalyptus Eucalyptus globules and clove Eugenia caryophyllus on albino rats. Polish J. Environ. Stud..

[86] Vijayasteltar L., Nair G.G., Maliakel B., Kuttan R., Krishnakumar I.M. (2016). Safety assessment of a standardized polyphenolic extract of clove buds: Subchronic toxicity and mutagenicity studies. Toxicol. Rep..

[87] Cortés-Rojas D.F., de Souza C.R.F., Oliveira W.P. (2014). Clove (Syzygium aromaticum): A precious spice. Asian Pac. J. Trop. Biomed..

[88] Hartnoll G., Moore D., Douek D. (1993). Near fatal ingestion of oil of cloves. Arch. Dis. Child..

[89] Slameňová D., Horváthová E., Wsólová L., Šramková M., Navarová J. (2009). Investigation of anti-oxidative, cytotoxic, DNA-damaging and DNA-protective effects of plant volatiles eugenol and borneol in human-derived HepG2, Caco-2 and VH10 cell lines. Mutat. Res. Toxicol. Environ. Mutagen..

[90] Doleželová P., Mácová S., Plhalová L., Pištěková V., Svobodová Z. (2011). The acute toxicity of clove oil to fish Danio rerio and Poecilia reticulata. Acta Vet. Brno.

[91] FAO/WHO (1982). Evaluation of Certain Food Additives and Contaminants: Twenty-Sixth Report of the Joint FAO/WHO Expert Committee on Food Additives [Meeting Held in Rome from 19 to 28 April 1982].

[92] Abdullah B.H., Hatem S.F., Jumaa W. (2015). A Comparative Study of the Antibacterial Activity of Clove and Rosemary Essential Oils on Multidrug Resistant Bacteria. Pharm. Biosci. J..

[93] Mostafa A.A., Al-Askar A.A., Almaary K.S., Dawoud T.M., Sholkamy E.N., Bakri M.M. (2018). Antimicrobial activity of some plant extracts against bacterial strains causing food poisoning diseases. Saudi J. Biol. Sci..

[94] National Center for Biotechnology Information PubChem Taxonomy Summary for TaxonomyBob 52462, Cuminum Cyminum. https://pubchem.ncbi.nlm.nih.gov/taxonomy/Cuminum%20cyminum.

[95] Singh R.P., Gangadharappa H.V., Mruthunjaya K. (2017). *Cuminum cyminum*—A Popular Spice: An Updated Review. Pharmacogn. J..

[96] Siow H.-L., Gan C.-Y. (2016). Extraction, identification, and structure–activity relationship of antioxidative and α-amylase inhibitory peptides from cumin seeds (*Cuminum cyminum*). J. Funct. Foods.

[97] Singh N., Yadav S.S., Kumar S., Narashiman B. (2021). A review on traditional uses, phytochemistry, pharmacology, and clinical research of dietary spice *Cuminum cyminum* L.. Phyther. Res..

[98] Rajput R.P.S., Paramakrishnan N., Gangadharappa H.V. (2021). Cumin (*Cuminum cyminum* L.) Seed. Oilseeds: Health Attributes and Food Applications.

[99] Animal Gourmet Comino, la Semilla del Sabor Virreinal. https://www.animalgourmet.com/2015/04/17/50saboresmexicanos-comino-la-semilla-del-sabor-virreinal/#:~:text=En%20M%C3%A9xico%20fue%20popularizada%20por,su%20sabor%20picante%20y%20acre.

[100] EI-kani M.H., Golmohammad F., Mirza M., Rowshanzamir S. (2007). Extraction of volatile oil from cumin (*Cuminum cyminum* L.) With superheated water. J. Food Process Eng..

[101] Iacobellis N.S., Lo Cantore P., Capasso F., Senatore F. (2005). Antibacterial Activity of *Cuminum cyminum* L. and *Carum carvi* L. Essential Oils. J. Agric. Food Chem..

[102] Bokaeian M., Shiri Y., Bazi S., Saeidi S., Sahi Z. (2014). Antibacterial activities of *Cuminum cyminum* Linn. Essential Oil Against Multi-Drug resistant *Escherichia coli*. Int. J. Infect..

[103] Sattari M., Bigdeli M., Derakhshan S. (2010). Effect of cumin (*Cuminum cyminum*) seed essential oil on biofilm formation and plasmid Integrity of Klebsiella pneumoniae. Pharmacogn. Mag..

[104] Saee Y., Dadashi M., Eslami G., Goudarzi H., Taheri S., Fallah F. (2016). Evaluation of Antimicrobial Activity of *Cuminum cyminum* Essential Oil and Extract against Bacterial Strains Isolated from Patients with Symptomatic Urinary Tract Infection. Nov. Biomed..

[105] Gupta A.D., Bansal V.K., Babu V., Maithil N. (2013). Chemistry, antioxidant and antimicrobial potential of nutmeg (Myristica fragrans Houtt). J. Genet. Eng. Biotechnol..

[106] Rehman J.U., Iqbal A., Mahmood A., Asif H.M., Mohiuddin E., Akram M. (2021). Phytochemical analysis, antioxidant and antibacterial potential of some selected medicinal plants traditionally utilized for the management of urinary tract infection. Pak. J. Pharm. Sci..

[107] Prakash E., Gupta D.K. (2014). Cytotoxic Activity of Ethanolic Extract of *Cuminum cyminum* Linn against Seven Human Cancer Cell Line. Univers. J. Agric. Res..

[108] Niu C., Gilbert E.S. (2004). Colorimetric Method for Identifying Plant Essential Oil Components That Affect Biofilm Formation and Structure. Appl. Environ. Microbiol..

[109] Gutmann L., Williamson R., Moreau N., Kitzis M.-D., Collatz E., Acar J.F., Goldstein F.W. (1985). Cross-Resistance to Nalidixic Acid, Trimethoprim, and Chloramphenicol Associated with Alterations in Outer Membrane Proteins of Klebsiella, Enterobacter, and Serratia. J. Infect. Dis..

[110] Johri R. (2011). *Cuminum cyminum* and Carum carvi: An update. Pharmacogn. Rev..

[111] Chacón-Vargas K.F., Sánchez-Torres L.E., Chávez-González M.L., Adame-Gallegos J.R., Nevárez-Moorillón G.V. (2022). Mexican Oregano (Lippia berlandieri Schauer and Poliomintha longiflora Gray) Essential Oils Induce Cell Death by Apoptosis in Leishmania (Leishmania) mexicana Promastigotes. Molecules.

[112] Bautista-Hernández I., Aguilar C.N., Martínez-Ávila G.C.G., Torres-León C., Ilina A., Flores-Gallegos A.C., Kumar Verma D., Chávez-González M.L. (2021). Mexican Oregano (Lippia graveolens Kunth) as Source of Bioactive Compounds: A Review. Molecules.

[113] García-Pérez E., Francisco Castro-Álvarez F., Alejandra Gutiérrez-Uribe J., García-Lara S. (2012). Revisión de la producción, composición fitoquímica y propiedades nutracéuticas del orégano mexicano* Revision of the production, phytochemical composition, and nutraceutical properties of Mexican oregano. Rev. Mex. Cienc. Agrícolas.

[114] Soltani S., Shakeri A., Iranshahi M., Boozari M. (2021). A review of the phytochemistry and antimicrobial properties of origanum vulgare l. And subspecies. Iran. J. Pharm. Res..

[115] Fimbres-García J.O., Flores-Sauceda M., Othon-Díaz E.D., García-Galaz A., Tapia-Rodríguez M.R., Silva-Espinoza B.A., Ayala-Zavala J.F. (2022). Facing Resistant Bacteria with Plant Essential Oils: Reviewing the Oregano Case. Antibiotics.

[116] Zapién-Chavarría K.A., Plascencia-Terrazas A., Venegas-Ortega M.G., Varillas-Torres M., Rivera-Chavira B.E., Adame-Gallegos J.R., González-Rangel M.O., Nevárez-Moorillón G.V. (2019). Susceptibility of Multidrug-Resistant and Biofilm-Forming Uropathogens to Mexican Oregano Essential Oil. Antibiotics.

[117] Xiao S., Cui P., Shi W., Zhang Y. (2020). Identification of essential oils with activity against stationary phase Staphylococcus aureus. BMC Complement. Med. Ther..

[118] Hadi Alkhafaji R.T., Jayashankar M. (2022). Physicochemical Properties and Inhibitory Effects of Oregano Oil against Uropathogenic. Pharmacogn. Res..

[119] Benbrahim C., Barka M.S., Basile A., Maresca V., Flamini G., Sorbo S., Carraturo F., Notariale R., Piscopo M., Khadir A. (2021). Chemical Composition and Biological Activities of Oregano and Lavender Essential Oils. Appl. Sci..

[120] Drissi B., Mahdi I., Yassir M., Ben Bakrim W., Bouissane L., Sobeh M. (2022). Cubeb (Piper cubeba L.f.): A comprehensive review of its botany, phytochemistry, traditional uses, and pharmacological properties. Front. Nutr..

[121] Akshita C., Vijay B.V., Praveen D. (2020). Evaluation of phytochemical screening and antimicrobial efficacy of Mesua Ferrea and Piper cubeba fruit extracts against multidrug resistant bacteria. Pharmacophore.

[122] Al-Shahwany A.W. (2014). Alkaloids and Phenolic Compound Activity of *Piper Nigrum* against Some Human Pathogenic Bacteria. Biomed. Biotechnol..

[123] Subbu Lakshmi S., Chelladurai G., Suresh B. (2016). In vitro studies on medicinal plants used against bacterial diabetic foot ulcer (BDFU) and urinary tract infected (UTI) causing pathogens. J. Parasit. Dis..

[124] Chaudhary V., Rk R., Chaudhary N., Sharma G. (2019). Bio-control of multiple drug-resistant uropathogens using medicinal plant extracts. Asian J. Pharm. Clin. Res..

[125] Takooree H., Aumeeruddy M.Z., Rengasamy K.R.R., Venugopala K.N., Jeewon R., Zengin G., Mahomoodally M.F. (2019). A systematic review on black pepper (*Piper nigrum* L.): From folk uses to pharmacological applications. Crit. Rev. Food Sci. Nutr..

[126] Kadosh Y., Muthuraman S., Yaniv K., Baruch Y., Gopas J., Kushmaro A., Kumar R.S. (2021). Quorum Sensing and NF-κB Inhibition of Synthetic Coumaperine Derivatives from *Piper nigrum*. Molecules.

[127] Srinivasan R., Vigneshwari L., Rajavel T., Durgadevi R., Kannappan A., Balamurugan K., Pandima Devi K., Veera Ravi A. (2018). Biogenic synthesis of silver nanoparticles using Piper betle aqueous extract and evaluation of its anti-quorum sensing and antibiofilm potential against uropathogens with cytotoxic effects: An in vitro and in vivo approach. Environ. Sci. Pollut. Res..

[128] Karadağ A.E., Demirci B., Çaşkurlu A., Demirci F., Okur M.E., Orak D., Sipahi H., Başer K.H.C. (2019). In vitro antibacterial, antioxidant, anti-inflammatory and analgesic evaluation of *Rosmarinus officinalis* L. flower extract fractions. South African J. Bot..

[129] González-Minero F.J., Bravo-Díaz L., Ayala-Gómez A. (2020). *Rosmarinus officinalis* L. (Rosemary): An Ancient Plant with Uses in Personal Healthcare and Cosmetics. Cosmetics.

[130] Younes M., Aggett P., Aguilar F., Crebelli R., Dusemund B., Filipič M., Frutos M.J., Galtier P., Gott D., Gundert-Remy U. (2018). Refined exposure assessment of extracts of rosemary (E 392) from its use as food additive. EFSA J..

[131] de Oliveira J.R., Camargo S.E.A., de Oliveira L.D. (2019). *Rosmarinus officinalis* L. (rosemary) as therapeutic and prophylactic agent. J. Biomed. Sci..

[132] Senanayake S.P.J.N. (2018). Rosemary extract as a natural source of bioactive compounds. J. Food Bioact..

[133] Nieto G., Ros G., Castillo J. (2018). Antioxidant and Antimicrobial Properties of Rosemary (*Rosmarinus officinalis*, L.): A Review. Medicines.

[134] Kloy A., Ahmad J., Yusuf U., Muhammad M. (2020). Antibacterial Properties of Rosemary (*Rosmarinus officinalis*). South Asian Res. J. Pharm. Sci..

[135] Li L., Li Z.-W., Yin Z.-Q., Wei Q., Jia R.-Y., Zhou L.-J., Xu J., Song X., Zhou Y., Du Y.-H. (2014). Antibacterial activity of leaf essential oil and its constituents from Cinnamomum longepaniculatum. Int. J. Clin. Exp. Med..

[136] Lagha R., Ben Abdallah F., AL-Sarhan B., Al-Sodany Y. (2019). Antibacterial and Biofilm Inhibitory Activity of Medicinal Plant Essential Oils Against *Escherichia coli* Isolated from UTI Patients. Molecules.

[137] Abdulhasan G.A. (2017). Synergism effect of rosemary essential oil and some antibiotic against *Escherichia coli* isolated from clinical samples. IOSR J. Pharm. Biol. Sci..

[138] Amirian F., Kazemi Pour N., Khoshroo S.M.R., Sayadi A., Karmostaji A., Mousavi S.M. (2017). Synergistic Effect and Antibacterial Activities of Extracts of Salvia and Rosemary Officinalis Against *Escherichia coli* Isolated from Clinical Urinary Tract Infection. Ann. Mil. Health Sci. Res..

[139] Beigomi M., Biabangard A., Rohani R. (2021). Evaluation of antimicrobial effects of Rosemary and Withania somnifera methanol extract prepared by ultrasound waveform on *Escherichia coli* biofilm isolated from urinary tract infection. Micro Env..

[140] Petrolini F.V.B., Lucarini R., de Souza M.G.M., Pires R.H., Cunha W.R., Martins C.H.G. (2013). Evaluation of the antibacterial potential of Petroselinum crispum and *Rosmarinus officinalis* against bacteria that cause urinary tract infections. Braz. J. Microbiol..

[141] Sienkiewicz M., Łysakowska M., Pastuszka M., Bienias W., Kowalczyk E. (2013). The Potential of Use Basil and Rosemary Essential Oils as Effective Antibacterial Agents. Molecules.

[142] Al Zuhairi J.J.M.J., Jookar Kashi F., Rahimi-Moghaddam A., Yazdani M. (2020). Antioxidant, cytotoxic and antibacterial activity of *Rosmarinus officinalis* L. essential oil against bacteria isolated from urinary tract infection. Eur. J. Integr. Med..

[143] Wawrysiuk S., Naber K., Rechberger T., Miotla P. (2019). Prevention and treatment of uncomplicated lower urinary tract infections in the era of increasing antimicrobial resistance—Non-antibiotic approaches: A systemic review. Arch. Gynecol. Obstet..

[144] Sabadash M., Shulyak A. (2017). Canephron^®^ N in the treatment of recurrent cystitis in women of child-bearing Age: A randomised controlled study. Clin. Phytoscience.

[145] Rechberger E., Rechberger T., Wawrysiuk S., Miotla P., Kulik- Rechberger B., Kuszka A., Wróbel A. (2020). A Randomized Clinical Trial to Evaluate the Effect of Canephron N in Comparison to Ciprofloxacin in the Prevention of Postoperative Lower Urinary Tract Infections after Midurethral Sling Surgery. J. Clin. Med..

